# Understanding Undergraduate Medicines Optimisation Education and Its Importance; a Realist Evaluation

**DOI:** 10.5334/pme.2450

**Published:** 2026-08-25

**Authors:** Richard Bodington, Joanne Reeve, David Hepburn, Matthew Morgan, Paul E.S. Crampton

**Affiliations:** 1 Health Professions Education Unit, Hull-York Medical School, University of York, Heslington, York, YO10 5DD, UK; 2 Hull-York Medical School, University of York, Heslington, York, YO10 5DD, UK

## Abstract

**Introduction:**

Problematic polypharmacy (PP) is associated with risks and burdens for patients, and waste for healthcare systems. To overcome this challenge, clinicians must be able to tailor clinical decisions to patients’ individual circumstances by integrating biomedical with biographical accounts of illness. This skillset has not been well defined or taught. Medicines optimisation (MO) is a key clinical activity in tackling PP and can exemplify the practical wisdom required for this integration. How and why MO education works is poorly understood. Our previous work, a realist review of published interventions, addressed this gap by explaining how undergraduate MO education could work but was limited by the thin descriptions available. Here, we develop and refine our explanatory model using primary data.

**Methods:**

This realist evaluation develops the programme theory from our review using primary data from individual realist interviews of clinical and educational MO experts. A realist configurational approach to analysis was employed. The study is conducted and reported in-keeping with RAMESES II standards.

**Results:**

Sixteen experts were interviewed; data were used to refine or refute our previous theorising. Analysis showed MO education which seeks to integrate interpretive practice into clinical decision-making must be sufficiently valued by stakeholders at all levels to garner the allocation of resources required for meaningful engagement with this education. Learners must feel they have permission to practice interpretive medicine when healthcare structures are organised to the contrary, this permission should be actively scaffolded by institutional and workplace context. A perception of critical mass within a community of practice may facilitate the required security in decision-making for ongoing practice.

**Discussion:**

We describe how and why undergraduate MO educational programmes can work to produce important outcomes in tackling the challenge of PP. We suggest how MO educational programmes may also act as practical gateways to routine interpretive practice in broader clinical areas.

## Introduction

People living with multiple long term health conditions (MLTC) are not well served by a rigidly guideline-led approach to disease management [1]. Such an approach may contribute to the growing challenge of problematic polypharmacy (PP- defined in Table 1) which is associated with risk and burden for patients and waste for healthcare systems [1,2,3,4]. Accordingly, there have been calls for a paradigm shift in approach to the healthcare of these patients [1,5,6]. These calls advocate for a focus on shared decision-making and practical wisdom to affect individualised, safe and effective changes to patients’ care using an interpretive, generalist understanding of illness (for definitions see Table 1) [1]. Yet the practical translation of this call into effective educational interventions has been challenging [7]. As a result, physicians still graduate medical school with heavily biomedical approaches to disease management and may struggle to apply interpretive approaches to clinical reasoning and decision-making.

**Table 1:** A glossary of terms used in this paper.


TERM	DEFINITION

**MEDICAL GENERALISM**	An illness approach that considers the patient’s whole health and well-being, not just their symptoms; it is holistic and adaptable [19]. It is a particular skill of physicians and can be practiced by all specialties, not only general practitioners [19,20].

**INTERPRETIVE PRACTICE**	The critical, thoughtful, professional use of an appropriate range of knowledges in the dynamic, shared exploration and interpretation of individual illness experience, in order to support the creative capacity of individuals in maintaining their daily lives [21].

**PROBLEMATIC POLYPHARMACY (PP)**	Polypharmacy denotes the use of ≥5 medications simultaneously; this can be appropriate. Problematic polypharmacy describes the situation where the intended benefits from the use of multiple medications are not realised [4].

**MEDICINES OPTIMISATION (MO)**	‘A person centred approach to safe and effective medicines use, to ensure people obtain the best possible outcomes from their medicines’ [9]. N.B. In North America this term may be conflated with medicines intensification. This is not how the term is used in this paper.

**DEPRESCRIBING**	‘The supervised withdrawal of potentially inappropriate medication; it is a planned, supervised process of dose reduction or the stopping of medicines that may be causing harm or conferring no additional benefit with the goal of managing polypharmacy and improving outcomes’ [2].

**MECHANISM**	In realist evaluation, underlying processes that are real but usually hidden, which operate in particular contexts to generate outcomes of interest [22].

**CONTEXT, (INTERVENTION), MECHANISM, OUTCOME CONFIGURATION (C(I)MOC)**	A heuristic, in the form of a statement or diagram that spells out the relationship between particular features of context, particular mechanisms and particular outcomes [22]. Intervention may be added to the configuration to form a CIMOC to avoid conflation between context and intervention.

**PROGRAMME THEORY (PT)**	A hypothesis, developed through realist research via primary or secondary data to explain how a programme works to produce outcomes [23].


The practice of medicines optimisation (MO) frequently involves complex, creative and nuanced clinical decision-making and it has been suggested that its education may act as a practical means through which learners can develop interpretive generalist capabilities [8]. MO describes the person-centred approach to safe and effective medicines use to ensure people obtain the maximum benefit for their medications, if they require them [9]. It encompasses the activities of both deprescribing and addition of medications within a whole person context [10]. MO is a key aspect of the care of people with MLTC; it is central to recognising causes of PP, such as prescribing cascades, and provides the means to address them [1]. Yet despite its importance, MO is frequently neglected in undergraduate medical curricula and many physicians graduate feeling they lack competence and a role in the task [11,12,13].

Limited research has examined undergraduate MO educational interventions. Educational interventions in the literature are predominantly single centre, stand-alone studies with significant heterogeneity in scope and outcome [8]. Thin descriptions of the interventions, make transferable learning challenging [8]. Our recent realist review is the only review of MO educational programmes in the undergraduate setting and the first attempt to clarify how and why such education works [8]. Our work described MO education as fundamentally sitting at a crossroads of illness perspectives between biomedical approaches to disease management and whole person interpretive understandings of illness that integrate biographical experiences of illness into clinical decision making. Our review noted that the clinical practice of MO involves routine integration of these two agendas for clinical decision making. Our analysis was limited by the thin descriptions and paucity of secondary data [8].

To address the limitations of the literature and to enhance our understanding of this important gap within medical education theory and practice, we next sought to add the expertise of front-line educators in this study to develop the programme theory (PT) from our preceding review. Our research question asked, in the experience of active medical educators, ‘what works, for whom, under what circumstances, how and why in MO education at the undergraduate level’. Our aim was to enhance the explanatory power of programme theorising from our prior realist review by developing it with experiential data from practitioners and educators on the ground.

## Methods

This study was designed, conducted and reported in keeping with the Realist And Meta-narrative Evidence Syntheses: Evolving Standards (RAMESES) II standards on quality and reporting standards in realist evaluation (RE) research [14,15].

### Rationale for using a realist evaluation approach

Realist evaluation refers to primary realist research; RE does not need to take the form of an evaluation of an intervention and can be used to clarify and describe phenomena, as in this study [15]. RE provides a useful approach to unpacking the inner workings of the ‘black box’ of complex interventions [16]. Other methodologies such as experimental study designs and traditional systematic reviews have, to date, been unable to provide transferable insight into the workings of the complex, context dependant interventions that prescribing and MO education programmes represent [17]. Realist researchers develop and analyse configurations of context-mechanism and outcome (CMOC) from data to develop and refine explanations of how and why outcomes happen [18].

### Description of subject under evaluation

Although MO is a policy priority in multiple countries, only a minority of medical schools provide specific MO and deprescribing education and a limited number of MO educational interventions within undergraduate curricula have been described and evaluated in the literature [8,11]. Many of these reports describe elective and stand-alone programmes delivered in single centres [8]. Interviewees in this study frequently described elements of MO education hidden, unlabelled or tokenistically included within broader clinical pharmacology and therapeutics (CPT) education. Many junior doctors graduate feeling they lack skills and understanding of their role in MO within the multidisciplinary team [12,13]. It has been suggested that junior doctors frequently graduate perceiving MO as primarily a task for pharmacists [12,13]. The views of educators on the ground have not been explored.

### Evaluation design description and justification

This study is a realist interview study. This study was conducted in keeping with the approach and conceptual framework of the seminal realist interview papers (Pawson and Tilley, Pawson, Manzano and Mukumbang) [24,25,26,27,28]. Subject experts and programme developers are apt to provide insight into the contexts and outcomes of interventions; expert stakeholders are particularly useful when evaluators are unclear on the nature of programmes, as in this case [27]. Expert realist interviews are well-placed to refine theories gleaned from the literature. This study’s scope was focused within the UK setting to be cognizant of the wider programme contexts and achieve a useful information power within a practical number of interviews [29]. The Hull York Medical School Research Ethics Committee approved the study protocol in February 2024 (HYMS approval number 23-24.36). Signed informed consent was obtained before the start of data collection.

### Recruitment process, sampling strategy and data collection methods

We completed a series of hour-long online realist individual interviews with experts in the fields of MO, polypharmacy and CPT education. Interviewees were recruited purposively based on their experience in MO/PP and CPT UG education, with attention to geographical and institutional diversity [27]. Recruitment was halted based upon an iterative perception of sufficient information power based on the specificity and quality of dialogue [29].

The study population included individuals with a steering committee role or practical experience in design of educational interventions in CPT, prescribing and/or MO. In keeping with realist principles, *a priori* inclusion and exclusion criteria were not applied [24,30]. Instead, potential interviewees were approached on the basis of their ability to contribute to the developing PT at that time; as such the sampling strategy was dynamic and adaptive. Potential interviewees were identified and approached via email as key authors in the field identified via the prior literature search, or as nationally prominent clinical educators with an interest in MO or prescribing educational interventions [8]. As such, interviewees consisted of health professionals with a mix of professional roles, often held simultaneously. Interviewees’ primary job roles included clinical educators, clinical education researchers and applied health researchers. All had a role in teaching.

Interviews were conducted, recorded, and transcribed via an online video conferencing platform (MS Teams, Microsoft, Redmond, WA, USA). The interview schedule was semi-structured and iteratively developed and adapted depending on the expertise of the interviewee and the requirements of the PT at that time (supplementary file 1) [14,15]. Interview transcripts were checked by RB against the audio recordings and corrected as needed for transcriptional accuracy before anonymisation. All transcripts required multiple minor amendments for transcriptional accuracy. The transcripts alone were used as primary data. Member checking of transcripts was offered to, and subsequently declined by, all interviewees. A summary of the findings of this study was fed back to all participants with comments invited. Minimal comments were received as a result of this feedback, none of which contributed to programme theorising.

A full interview transcript is provided in Supplementary File 1 as an illustrative example of the interview schedule used, the quality of dialogue and the use of realist interview technique.

### Data analysis

Data analysis was iterative and concurrent with data collection to allow adaptability in sampling [14,28]. All analysis was theory driven with evidence compared to our initial programme theory (iPT) from our prior review (supplementary file 2) and the developing PT from this study [14].

Transcripts were analysed using the realist configurational analysis method described by Gilmore and colleagues [31]. Transcripts were imported into a qualitative data analysis software package (NVivo, Lumivero, Denver, CO, USA). A code book was created with nodes based on the 19 elements of the iPT with iterative memos linked to each node for transparent documentation [31]. The iPT was developed using interview data, with elements being refined or refuted and subsequently expanded or collapsed into each other. CMOC were not lifted from the transcripts verbatim but synthesised through analysis and integration of a variety of responses. Iterative sharing of memos between the research team acted to check inferences and identify conceptual avenues to explore. The formal theories of experiential learning theory and transformative learning theory were used to underpin theorising throughout the review and interview study [32,33]. These formal theories were used to provide widely accepted and relatively transparent underpinnings to our steps of retroductive inference during the studies.

Once all interview transcripts had been analysed and CMOCs developed, the CMOCs were grouped according to demi-regularities within them. This process led to three CMOC clusters which were used to develop and explain the overall PT. These clusters were: the contextual drivers of value, prioritisation and engagement with MO education, the resource and response mechanisms triggered by elements of interventions within MO educational programmes, and explanations pertaining primarily to educational outcomes. The CMOC clusters were expanded and explained in prose with the aid of the afore mentioned memos and synthesised into partial-PT diagrams for clarity. The combined explanations and partial-PT diagrams were then used to develop an overall PT diagram as a summary of the explanations and to communicate the linkages between them.

### Reflexivity

Analysis was undertaken from a realist ontological and epistemological perspective. The research team was comprised of four clinical academics (all physicians, one in primary care) and two non-clinical academics. Five individuals were primarily medical educators with the remainder a primary care researcher. Three were experienced in RE and one an expert in polypharmacy. Reflexivity was checked regularly using the matrix proposed by Downey and colleagues, transparent documentation of PT development through memo documents, and triangulation with the MO literature and substantive learning theory [34].

## Results

### Details of participants

Sixteen participants were interviewed (see Table 2 for details). No participants withdrew from the study, and all transcribed data was analysed.

**Table 2:** Details of interviewees. Please note that most interviewees had split job roles and taught healthcare professionals alongside research, administrative or policy roles.


CATEGORY	DETAILS

**NUMBER OF PARTICIPANTS**	16

**MEDIAN INTERVIEW DURATION**	55 minutes

**GENDER DISTRIBUTION**	Male: 9, Female: 7

**GEOGRAPHIC REPRESENTATION**	All devolved nations of the UK (England, Scotland, Wales, Northern Ireland)

**PROFESSIONAL BACKGROUND**	Pharmacists: 6, Physicians: 10

**UNIVERSITY AFFILIATIONS**	12 UK universities represented; 10 interviewees held professorial chairs

**ORGANISATIONAL AFFILIATIONS**	The majority of interviewees held senior committee membership: NHS England, National Institute of Health and Care Research, Royal Pharmaceutical Society, British Pharmacological Society, Prescribing Safety Assessment, Medical Schools Council, and others.

**EXPERTISE AREAS**	Prescribing safety, polypharmacy, medicines optimisation (including deprescribing and systematic medication reviews, within clinical practice or education), clinical reasoning, behaviour change, interpretive generalist medical practice, clinical pharmacology and therapeutics education, assessment in medical education.

**PROFESSIONAL LEARNER GROUPS TAUGHT**	Physicians/medical students: 12 Pharmacists/pharmacy students: 4 Allied health professionals: 6 Interviewees frequently taught >1 professional group.


### Main findings

**Figure 1:**
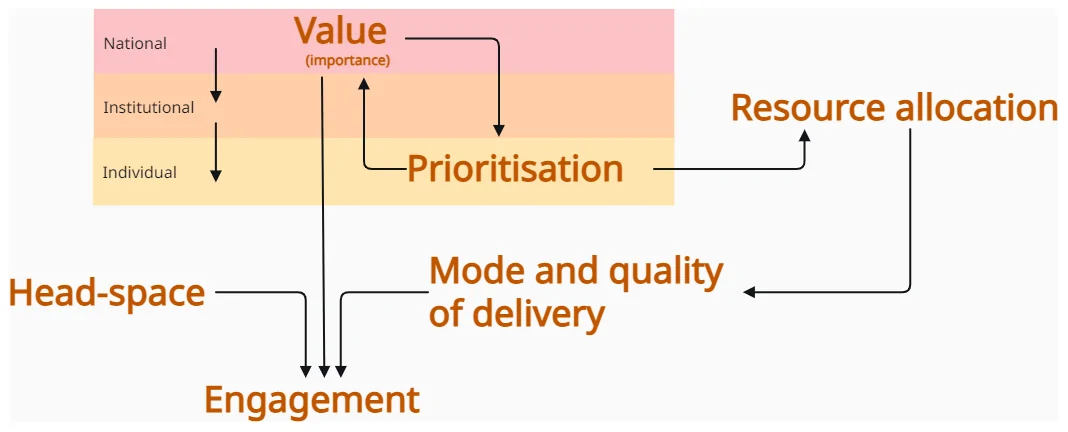
Partial programme theory #1: Prioritisation, perceptions of value (that is importance) and engagement. Black arrows suggest how one element might influence another. The national context will influence the institutional context which will influence the individual learner context.

**Figure 2:**
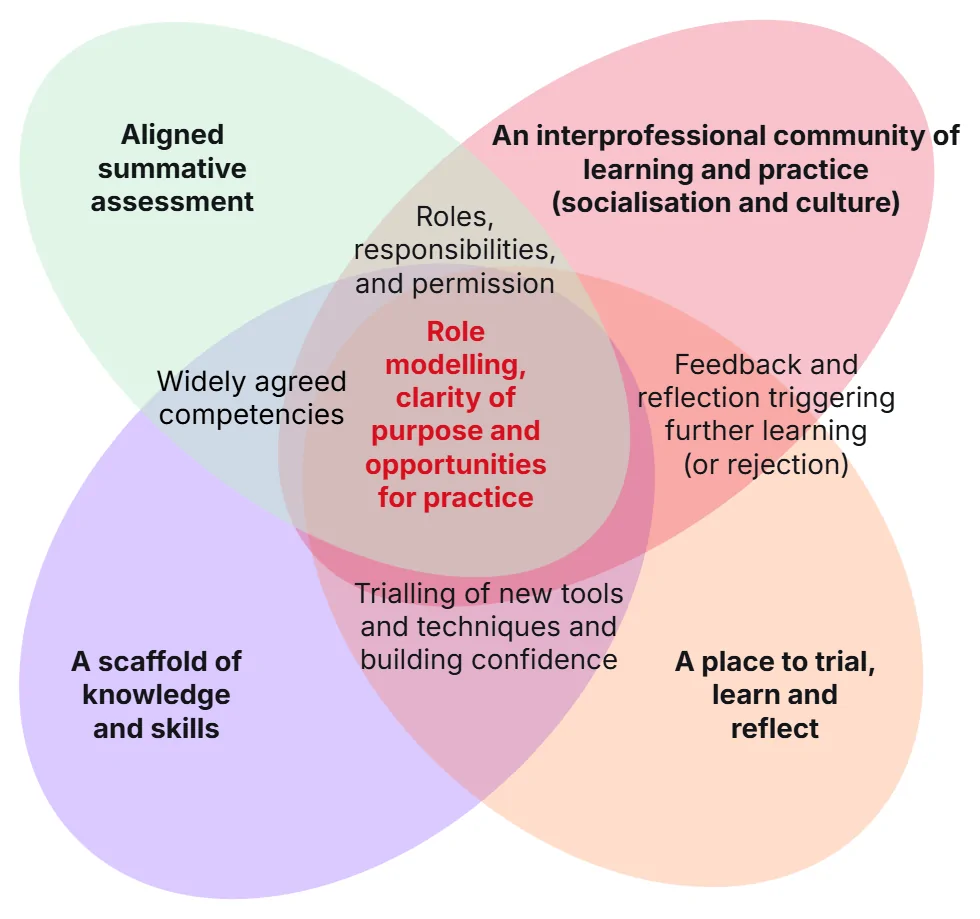
Partial programme theory #2: Prerequisites, a place to trial and reflect and a community of practice. Each oval represents a programme intervention element; the areas of overlap represent resource or response mechanisms.

**Figure 3:**
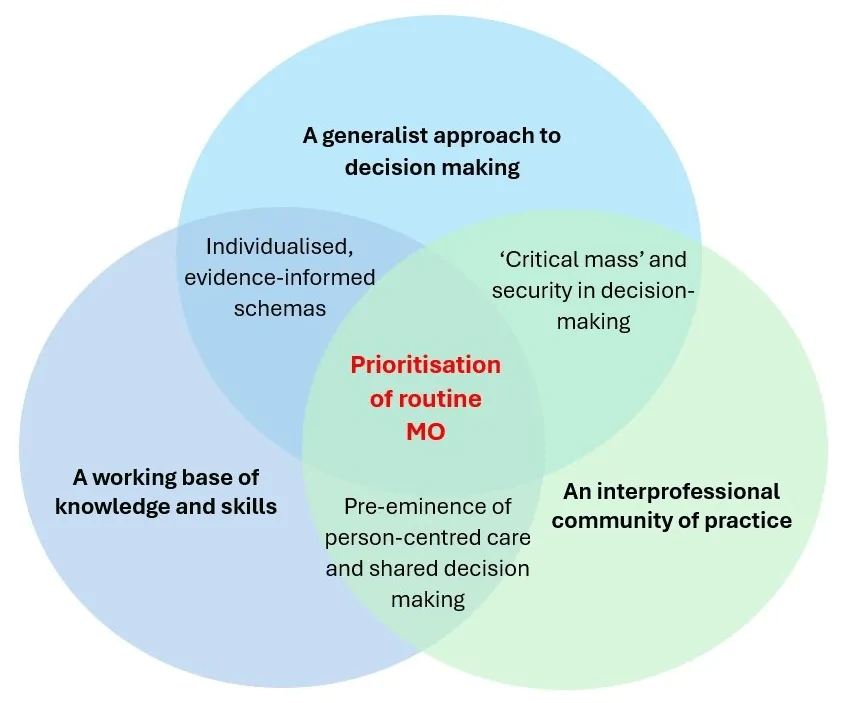
Partial programme theory #3: An interprofessional generalist community of practice prioritising routine medicines optimisation. The circles represent broader outcomes with more specific outcomes at the areas of overlap.

**Figure 4:**
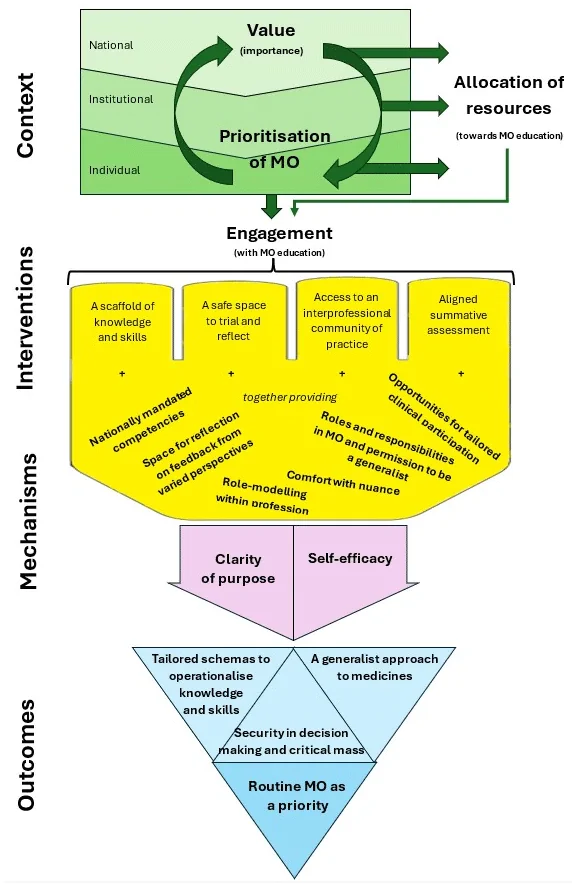
Diagram of the overall programme theory explaining the generation of outcomes from MO educational programmes at the undergraduate level. The diagram uses all 35 CMOC en masse and is designed to demonstrate the links between the three partial programme theories (Figures 1, 2, and 3). Mechanisms within the yellow area are those provided by the intervention’s resources. The mechanisms in lilac are learners’ responses to those resources. MO: medicines optimisation.

Analysis generated 35 CMOC that described three key elements of MO educational programme functioning (supplementary file 3). These three elements are: 1) the need to address prioritisation and perceptions of value towards MO because of their effect on stakeholder engagement with the education (Figure 1), 2) the requirement for prerequisites: a place to trial and reflect and a community of practice (Figure 2) and 3) learners’ perception of membership within an interprofessional generalist community of practice prioritising MO (Figure 3). These three key elements (or partial-PT) integrate to describe an overall theory of MO educational programme functioning in undergraduate medical curricula (Figure 4). We start by explaining the three key elements separately before describing how these integrate into an overall theory.

#### 1. Prioritisation, perceptions of value towards MO and stakeholder engagement (a partial-PT synthesizing CMOCs #3-12)

Our analysis demonstrates the importance of prioritisation and perceptions of value towards MO for stakeholders’ engagement with the education. Stakeholders include learners, institutional programme directors and educational policy decision-makers within bodies such as professional regulators. Engagement with MO education may be driven by stakeholders’ perceptions of available headspace for, and the value of, MO. These perceptions affect stakeholders’ decision-making around prioritisation of the subject. Prioritisation subsequently effects resource allocation and the mode and quality of education delivery (Figure 1).

Our analysis of experienced educators’ responses highlights that the perception of the value, or importance, of MO education promotes its prioritisation within curricula and the resources allocated towards it. Context affects perceived value and prioritisation in a cycle. For example, national prioritisation will promote institutional perception of MO education value (CMOC #3). Prioritisation in turn influences resource allocation. For example, if at the national level professional regulators prioritise MO ability and mandate MO competencies in medical graduates, and its inclusion within summative assessments, institutions are likely to allocate greater resources towards its education (CMOC #7, 8, 12).

***4-252 (physician, undergraduate educator, national assessment lead):** For example in the MLA (medical licensing assessment) and in the prescribing safety assessment and such assessments like that where we’ve got a national sort of collaboration in terms of what we’re going to assess and what’s going to be in there, if we have that kind of leverage to get medicines optimisation within there in one way or another, that would push all medical schools to make sure they addressed it in a better way*.

At the institutional level, if medical schools allocate curriculum and placement time, faculty and other resources toward MO, learners are liable to value the skill more highly (CMOC #7). If learners value MO, they are more likely to engage with the education and allocate it the necessary time and mental resources (CMOC #6). The visibility of MO in medical programmes contributes to this cycle of prioritisation and value; engagement with MO is hampered by the fact that it is often hidden within broader prescribing competencies (CMOC #6). The assessment of the often-nuanced practice of MO via formats such as multiple-choice questions (MCQ), strongly incentivised by the wider drive for reliability in assessment, may threaten its meaningful integration into summative exams and therefore hamper engagement due to learners’ assessment-oriented approach to learning (CMOC #4 and 11).

***5-16 (physician, undergraduate educator, polypharmacy expert):** I’ve been asked to write questions on polypharmacy in the past for MCQs and it’s a bloody nightmare because the questions you ask are pointless. You can’t do that with polypharmacy and with uncertainty, you just can’t. It just does not fit the bill. So, the questions you end up asking are just not actually addressing, you know, anything particularly useful*.

Institutional resource allocation will dictate the format and quality of MO programme delivery and the availability of opportunities for students to learn and repeatedly apply skills learned, which will in turn affect engagement. Excellent classroom or simulated teaching without the resources to ensure opportunities for clinical MO experience may hinder learner engagement (CMOC #21).

Competing interests for headspace amongst actors at all levels may adversely affect engagement despite their perceptions of value in MO and the mode and quality of MO education delivery. The perception of available headspace may be influenced by the perceived or actual dissonance between the principles of MO education and the rest of the medical programme and wider clinical practice (CMOC #9, 10, 14 and 36). For example, learners at an institution that already centralises the principle of individualisation of care may feel that they have more headspace for MO because of this alignment and can therefore engage better with it. The interviewee quote below explains how learners may experience MO education, particularly on clinical placement, if this alignment is not addressed.

***4-12 (physician, postgraduate educator, generalism expert):** We’ve designed a system that’s so obsessed with standardising care, with reducing everything down into little units that we can standardise and push through, ideally as quickly as possible, that actually we prevent the rats on the wheel from delivering anything close to those people standing there, saying ‘you need a personalised optimisation of medicines’. Great, those two are incompatible*.

This partial-PT refines the contextual elements of our review’s PT. The interviewees’ practical experience of the challenges facing those attempting to develop education to tackle clinical complexity and nuance (which the interviewees unanimously agreed MO education was an exemplar of), greatly enriched the thin contextual descriptions gleaned from the literature. The challenges of increasing standardisation in clinical care and educational culture, financial challenges, practicalities of programme and assessment design and the difficulties in highlighting foundational, longitudinal concepts within curricula, were all gleaned from interviewees’ responses rather than the literature.

#### 2. Prerequisites, a place to trial and reflect and a community of practice (a partial-PT synthesizing CMOCs #1, 2, 13-15, 17, 18, 20-27, 29-31, 33, 36, 38, 40)

Our second element focusses on the components needed to deliver an MO educational intervention. Our analysis describes four elements of MO educational interventions needed to trigger learner responses that increase the likelihood of achieving programme outcomes described in section 3 (Figure 2). These intervention elements are: 1) aligned summative assessment, 2) a perception of an interprofessional community of learning and practice, 3) a place to trial and learn, and 4) a scaffold of knowledge and skills. These components provide learners with five resources: 1) an appreciation of professional roles regarding MO, 2) permission to undertake MO, 3) opportunities for practice, 4) role models, and 5) provision of feedback as a trigger for further learning. Where these resources coalesce two responses may be triggered in learners as key drivers of programme outcomes: self-efficacy and clarity of purpose (Figures 2 and 4)(CMOC #33).

Three of these intervention elements were present in the PT of our review (aligned summative assessment, a scaffold of knowledge and skills and a place to trial and learn). Their importance was affirmed by the interviewees; subsequent discussion allowed an exploration of the mechanisms they may trigger to a greater depth than was possible in the review. The new element, ‘an interprofessional community of learning and practice’, was developed through a merging and expansion of the review’s PT elements of ‘interprofessional clinical experience’ and ‘feedback in small interprofessional groups’.

Interviewees did not feel that lack of knowledge represented a major barrier to the practice of MO by junior doctors but did accept its presence in a coherent PT (CMOC #23). Evidence-based frameworks for approaching the task of MO may provide security for learners and educators (CMOC #22). In the education of medical students specifically, implicit frameworks for approaching MO utilising generalist principles focussing on patient priorities and goals may free-up headspace in learners and provide clarity as to its purpose (for example, use of the Bristol Medication Review Model as opposed to Beers’ Criteria)(CMOC #33) [35,36,37]. The use of extensive explicit frameworks and protocolisation of MO practice may risk the disengagement of this learner group, in part due to perceived professional norms regarding physicians’ attitudes to protocolisation (CMOC #31).

***5-16 (physician, undergraduate educator, polypharmacy expert):** (discussing implicit medication review models) As we say, this is something you should use flexibly to suit your own needs, and it’s there as a primer, as a prompt for people, and I hope that then reduces cognitive load by (allowing the learner to think) ‘oh, here’s something I can just flip back to every now and again. I don’t have to do everything on it’. It’s just a helpful reminder. I think the danger is if people do try to do everything on it, which is not the point of these things*.

Instead of requisite knowledge, MO practice was felt more contingent on appropriate skills (in clinical reasoning and shared decision-making) and attitudes (in person-centred, interpretive practice and generalism) in learners. Accordingly, an educationally safe space to learn, trial and reflect was seen as a key resource to build learner confidence in the clinical use of these complex models of practice (CMOC #22 and 25). Additionally, for the triggering of emotion, prompting of reflection and consolidation of learning, MO programmes require sufficient resources to deliver regular, meaningful exposure to MO scenarios in real patients (CMOC #15, 21 and 27). Medical students need to regularly observe MO practice in working doctors to trigger a perception of professional expectation in the task and its relevance to their future roles (CMOC #2, 14 and 17). It is likely that substantial resources will be required to develop staff and clinical placements to ensure learners regularly experience MO practice aligned with classroom and simulated sessions (CMOC #13). Failure to undertake this development may result in MO programmes being perceived as dissonant with clinical practice and therefore being rejected by learners (CMOC #36).

***4-25 (pharmacist, clinical MO researcher):** I think you’d want to almost cherry pick a little bit so they (learners) can see them (doctors) doing this as part of their day job, so you’ve got that kind of role recognition again that would reinforce that as well, right. You know ‘these people are really senior in my profession, they’re doing it, right I should definitely be doing it’. ‘And now linking what I’m doing at university, I can see now how that’s working out in practice’*.

Versus:

***4-25:** I guess there is a risk that if we send them out with some placement objectives about doing some of this stuff and then they then don’t see it happening in practice, or people in practise saying ‘Oh no, that’s far too risky. Leave it alone’ or ‘we don’t have time to do that’ or ‘that’s not a priority’…They might find that, obviously counterproductive*.

Permission to undertake MO and make outside of guideline, interpretive decisions according to patient priorities was raised as a key mechanism in successful programmes (CMOC #38). As part of clinical exposure, interviewees felt effective MO programmes needed to facilitate access to an interprofessional community of learning and practice that values and practices MO as a key aspect of their clinical work (CMOC #20) [38]. A relationship with, rather than observation of, this community, particularly combined with regular interprofessional group feedback, discussion and reflection, were felt to increase the likelihood of triggering this perception of permission (CMOC #24, 38, 40). Thus, whilst permission is perceived by learners as an internal response, it is scaffolded by the context actively created by their educational institutions. The formation of this new community PT element from the review’s elements of ‘interprofessional clinical experience’ and ‘feedback in small interprofessional groups’ was felt necessary to reflect the frequently concurrent nature of experience and feedback and to incorporate interviewee’s explanations of community and its associated mechanisms.

***5-16 (physician, undergraduate educator, polypharmacy expert):** If you have a safe space to share ideas and to recognise that there might not be a right and a wrong way to do this, and indeed that you might be potentially going against the norms. I think that that potentially does work*.

The final intervention element important for effective MO programme design, a strong theme throughout the interviews and in the literature, is aligned summative assessment as a driver of appropriately nuanced learning via the value-prioritisation cycle of partial-PT #1. Without this intervention element the other three elements may be insufficient to trigger the required engagement (CMOC #7, 8, 12). Interviewees concurred with findings in the literature that inappropriately simplistic assessment may drive learning in a direction not applicable to meaningful clinical MO practice [39]. They also agreed that formative only assessment may be perceived by learners as denoting the low value of MO [40].

#### 3. An interprofessional generalist community of practice prioritising routine medicines optimisation (a partial-PT synthesizing CMOCs #22, 25, 34, 35, 39, 40, 42)

Our third element focusses on the outcomes from MO educational programmes. Our interviewees highlighted three outcomes that may result from MO education at the undergraduate level which facilitate the prioritisation of MO as part of routine clinical practice. These outcomes are: 1) a generalist approach to decision making (a finding from our review which was confirmed and expanded in this analysis) and two new outcomes, 2) a perception of membership within a supportive interprofessional community of practice, and 3) a flexible working base of knowledge and skills in MO (Figure 3). Membership of an interprofessional community of MO practice may act as an element of the educational intervention (as discussed in section 2) or as an outcome depending on the context-mechanism-outcome configuration.

Our interviewees agreed with the findings from our review by suggesting that lack of biomedical knowledge is not the greatest barrier to the practice of MO. Safe and effective MO practice requires knowledge of clinical pharmacology, but alone this is insufficient. For sustainable clinical practice, interviewees suggested that this knowledge should be integrated with skills and appropriate attitudes in shared decision-making and interpretive, person-centred consultation models. Flexible, implicit MO models were seen as useful quality assured tools to guide this integration (for example, the Scottish Polypharmacy Guidance or the Bristol Medication Review Model) (CMOC #22) [35,41]. A working base in these knowledge and skills, which learners can use flexibly and tailor to their individual needs, is required for a consilient explanation of the workings of MO educational programmes (CMOC #25). Others have already developed competency frameworks for MO curricula and it was not the aim of this study to replicate these efforts [42]. Accordingly, interviews were directed away from a more granular understanding of knowledge outcomes and towards areas unexplored by other researchers. We direct readers seeking a granular understanding of undergraduate MO competencies towards these documents [42].

An interpretive generalist approach to clinical decision making was widely seen as a key enabler of the tailored optimisation of medicines (CMOC #34 and 39).

***4-12 (physician, postgraduate educator, generalism expert):** Medicines optimisation is about taking it even further back than thinking about medicines and it’s into advanced generalist clinical reasoning. Clinical decision making. Interpretation of illness and so on and so forth*.

Interpretive generalist practice also encourages learners to appreciate the patient as a whole person in their context. The clarity of purpose that a focus on the individual and their priorities facilitates was seen by interviewees as an enabler of clinical decision making within the maze of complexities and uncertainties intrinsic to MO practice in multimorbid patients (CMOC #35). This important link between generalism and clarity of purpose was absent from our review analysis.

**5-16 (*physician, undergraduate educator, polypharmacy expert):*** *The point is, of course, we don’t really know how to deal with complexity, uncertainties. So the easiest thing to do is to ask the patient what matters most to them and use that to steer your way through what is a very difficult maze of problems*.

Interviewees suggested that where the outcomes of a generalist approach to healthcare and a learners’ perception of inclusion within an interprofessional community of MO practice come together learners may find security in decision making and the perception of critical mass (CMOC #40, 42). The term ‘critical mass’ is used to denote the perception amongst practitioners that they are acting in the same manner and direction as their colleagues, that there is momentum behind their collective action, and that once critical mass is achieved it is easier to undertake the task than not (i.e. easier to optimise a patient’s medicines than to be one of the few who does not). The confidence and security engendered by this perception of critical mass within a community of practice may allow learners to overcome the inertia, and at times fear, inhibiting the routine optimisation of medicines that learners may otherwise encounter in the workplace (CMOC #22 and 34). One of our interviewees, an educator predominantly of non-medical prescribers, summarises these two educational outcomes in saying:

**5-2 (pharmacist, postgraduate educator):** *But I think also in terms of confidence to deprescribe, I think if you know that your line of thinking is aligned with what the person wants, and what a number of your colleagues is thinking, you’re more likely to be taking the step to discontinue a preventive treatment than if you feel like you’ve got to make a decision alone*.

Analysis of interviewees’ responses suggests that where MO educational programmes facilitate in learners a generalist approach to decision making, perception of inclusion within an interprofessional community of practice and an interpretive knowledge and skills base, tailored to the individual, the likelihood of routine MO is maximised.

#### Overall programme theory

Figure 4 consolidates the three partial-PT of MO programme functioning described above into a single overall PT diagram. It utilises all 35 CMOC to demonstrate the connections in explanations primarily concerning context, with those of the intervention itself, and its educational outcomes. The figure provides no new explanatory links between context, mechanism or outcome over those laid out in the explanations of the preceding three sections but instead summarises them diagrammatically.

Our analysis of the interview data with that derived from our review suggests that the national, institutional and individual contextual drivers of the value of MO influence its prioritisation. Prioritisation directs resource allocation towards the education. The combination of prioritisation and resource allocation are liable to strongly influence engagement with the education. Engagement is seen as pre-requisite to programme effectiveness. Four intervention elements within the design of MO programmes (1. A scaffold of knowledge and skills, 2. A safe space to trial and reflect, 3. Access to an interprofessional community of practice and 4. Aligned, nuanced summative assessment) may provide the resources that increase the likelihood of learners achieving the intended educational outcomes. These resources include an appreciation of roles and responsibilities towards MO, permission to act according to generalist principles, provision of role models and opportunities for clinical participation and feedback. These resources may trigger in learners the clarity of purpose and self-efficacy ultimately needed to make MO a priority as part of routine clinical work. This outcome may be facilitated through the coalescence of three related outcomes: 1. Development of a generalist approach to decision-making, 2. A working knowledge and skills base, tailored to the individual, and 3. A perception of security in decision-making within a community of practice.

## Discussion

### Summary of findings

Our findings describe the key elements of MO education grounded in generalist, interpretive medical practice. Our findings build on and extend our review’s PT by adding depth and detail through the integration of primary data from interviews of educational and clinical experts in MO. Although many elements of the refined PT presented in this study aligned well with the secondary data, there are several key aspects, developed through insight from the primary data, that did not feature prominently in the literature. The first of these is the emphasis on learners’ inclusion within a community of professional practice as a resource within the educational intervention as well as a programme outcome. This element of programme functioning brought together the review’s elements of interprofessional clinical experience, feedback in small interprofessional groups, headspace, roles and self-efficacy (supplementary file 2). The refined element of an interprofessional community of practice appreciates the concurrent nature of the previously described elements and links them more clearly to the outcomes of security, critical mass and permission needed for MO practice. The insights of our interviewees also facilitated a far greater appreciation of the contextual challenges facing MO education than was gleaned through the literature. Interviewees’ experiences of the trend towards standardisation of experience and assessment in medical education disincentivising engagement with interpretive MO education, and the competing demands on the headspace of national and institutional decision-makers were not prominent in our previous PT. Our refined PT, summarised in Figure 4, incorporates these insights with those from the literature to develop a richer explanation of how and why MO educational programmes work or fail to work.

The predominance of guideline-led care has contributed to the increasing challenge of PP in most of the developed world [1,43,44,45,46,47]. Guideline-led practice emphasises what the clinician knows, rather than how they use it; this focus creates challenges for managing PP. The interpretive, generalist practice of MO is a key method of tackling this challenge at the patient level and graduating physicians must be competent in this activity. An understanding of how MO education works has been described as an ‘educational imperative’ and our work uniquely describes how this can be achieved [39]. We describe the workings of MO educational interventions in the undergraduate medical setting by identifying key elements of educational context, programme interventions and outcomes and link them to the hidden mechanisms they trigger. In doing so we provide explanation as to ‘what works in undergraduate MO education, for whom, under what circumstances, how and why’ for the benefit of educators in the field.

We suggest that MO can be seen as a case-study for medical education requiring practical wisdom for the applied integration of the biomedical and interpretive perspectives of illness for clinical reasoning and decision-making. MO education may act as a gateway to routine interpretive practice for medical students, particularly for learners requiring demonstration of the clear clinical application of education to drive their engagement. MO education can display the benefits for patient care from this integration, which may be less explicit in other forms of person-centred education. We assert that understanding MO education may potentially provide transferable insight for the broader workings of educational interventions tackling these complex inter-related themes of practical generalist wisdom and decision making based on the integration of apparently dissonant illness perspectives. A summary of transferable insights from this study is presented in Table 3.

**Table 3:** Transferable insight resulting from an understanding of the workings of MO educational interventions at the undergraduate level.


INSIGHT

**1. Education seeking to integrate interpretive principles into decision-making embedded in the biomedical approach must be valued by learners to garner the engagement necessary for learning** - Learner perceptions of value/importance may be driven by the prioritisation of the education by their institution, which in turn will be driven by national prioritisation of the subject. National and institutional prioritisation decisions are likely to impact on educational engagement. - Learner perceptions of the value of education may also be enhanced by an appreciation of professional roles and responsibility towards the subject, its inclusion within appropriately aligned summative assessments, and clarity as to the purpose and clinical benefits of the education.

**2. Education seeking to enable students to integrate interpretive medical practice principles with the biomedical agenda should be sufficiently aligned with the culture experienced within educational institutions and the workplace so as not to be rejected by learners** - The achievement of such alignment may require significant development of clinical placements to ensure observed clinical practice is not perceived as dissonant from classroom education.

**3. Learners need to feel they have permission to practice according to interpretive principles when healthcare structures may be organised to the contrary** - The provision of this permission may be a key component of effective educational interventions aiming to develop interpretive practice. - Within their community of practice, a *perception of critical mass* may facilitate learners’ *perception of permission and security in decision making*. - Perception of permission may be enhanced by a focus on the *professional identity of the physician* as an interpretive, creative, boundary-spanning decision-maker. - Feedback on practice in small interprofessional groups, meeting regularly with relational continuity, may be a key intervention component to allow the reflection on concrete experience and assisted sense-making required to facilitate these three mechanisms. - Permission as an internal learner response is the product of an institutional and wider context that provides a pedological, epistemological and socio-cultural scaffolding to this permission. Institutions should consider their responsibility in explicitly creating this context.


### Comparison with existing literature

To our knowledge there have been no interview studies published exploring the settings or structures of MO or deprescribing education in the undergraduate medical setting. One focus group-based study exploring perceptions of deprescribing education in a mixed group of healthcare students, including approximately one third medical students, was conducted in the US [48]. Students in this study perceived a need for simulation, in particular simulated patient encounters, as a means of practicing deprescribing skills prior to clinical exposure [48]. The central importance of interprofessional clinical experience and collaboration, along with the appreciation of the benefit of understanding interprofessional roles regarding deprescribing, was also voiced by learners in keeping with our own work [48]. The study employed thematic analysis, used export coding to ascertain learner’s perceptions of educational need, and did not attempt to explore context or other aspects of causation; therefore, further parallels from the work cannot be drawn. To our knowledge the views of educators have not been explored in this or other interview studies.

Our realist review synthesised existing related work in the field of MO and made explicit alignments and contrasts [8]. The PT resulting from that review formed the starting point for the development of the PT of this study; as such reference can be made to comparisons between the literature, the preceding review and the work of this study. Turk and colleagues, in their large NIHR funded realist review of deprescribing interventions in clinical practice suggested that shared decision making, a multidisciplinary approach, the development of trust and learning from patient follow-up were potential interventions to support deprescribing [49]. Our findings are aligned with these strategies; we suggest why and how such strategies may be of value in the undergraduate setting and link them with contexts and outcomes of specific interest in the undergraduate setting. Our findings are aligned with Turk’s final PT on the core elements supportive of tailored prescribing; supportive infrastructure, shared explanations of medications with patients and a trail and learn approach [49]. We suggest the educational intervention elements that may create this perception of this supportive infrastructure, that may help learners developing abilities to explore shared explanations of medicines, and that provide opportunities for iterative cycles of experiential learning. We feel this alignment adds to the external validity of our PT. During this project discussions were also had with members of the Farrell group, an internationally prominent group of Canadian deprescribing expert educators. Our work is aligned with the themes running through their recommendations on a curriculum framework for an interprofessional approach to deprescribing [50].

Our review synthesised secondary data suggesting that student-run clinics may be a promising format through which to deliver the clinical aspects of MO education programmes [8]. These clinics may provide a practical setting to trigger several of our PT’s resource mechanisms such as opportunities for tailored clinical participation, role modelling, feedback from varied (interprofessional) perspectives, and appreciation of professional roles and responsibilities (Figure 4). These attributes are drawn from, and thus aligned with, the literature on student-run clinics [51,52,53,54,55].

Our work highlights membership within an interprofessional community of practice that prioritises MO as an important outcome from undergraduate MO education. The work of Reinders and others on the development of interprofessional identity has synergy with this aspect of our PT [56,57,58,59]. We align ourselves with the position that IPE should not focus narrowly on interprofessional competencies (i.e. knowledge, skills, behaviours and attitudes related to interprofessional collaboration) but should be concerned with interprofessional socialisation and the development of shared purpose and a sense of belonging (57, 58). MO is globally acknowledged to be an interprofessional activity (9, 50). We concur with the supposition that separate and clear professional and interprofessional identities are both required within interprofessional teams tackling complex clinical problems such as MO [56]. Our realist work and that of the interprofessional identity literature are aligned in our interest of the contextual triggers of these social identities [57].

### Strengths and limitations of the evaluation

The findings of this study are novel and address an important gap in our understanding of the theory and practice of medical education. Design and delivery of effective MO educational programmes have been described as an ‘educational imperative’ because of their importance in tackling the growing challenge of PP and, we argue, potential to offer broader transferable insights [39]. This study was designed and delivered in keeping with the quality and publication standards for RE published by the RAMESES II group (14, 15). Whilst we accept that our data is only a partial representation of reality, we suggest that this study has sufficient information power to provide useful insight for educators [29]. The study applied theory from the preceding review throughout and by building on this data and applying it to the design of interview schedules, the information power resulting from the dataset was enhanced [29]. By cross-referencing and building upon the literature the study also addresses the potential for skewed theorising that may result from the use of subject experts alone as a data source for PT development [8]. The study utilised transparent methods for PT development; all analysis, including CMOC drafting, concept development and theorising were captured electronically using memos linked to each PT node [31]. As a result, decisions, inferences and links between documents are explicit and can be audited. The work does not proport to be a final product and should instead be considered a contribution to a complex puzzle. The study PT will be subject to subsequent realist research and testing planned by our group.

This study is limited in several ways. Owing to the incomplete coverage of MO education in UK medical schools and the variation in the roles of interviewees (i.e. not all interviewees were directors of CPT programmes in medical schools), the interviews were not carried out in a case-based fashion. Many medical schools do not specifically teach MO, therefore interviews involved middle range theorising on the participant’s own experience with relevance to the CMOCs and PT elements put to them. Accordingly, opportunities for exploration of specific elements of context and the emergence of demi-regularities by cross-case analysis were missed. We were unable to explore in this study how this education works differently in different groups of learners or across institutional or clinical contexts. Our PT was developed primarily in relevance to medical students, accordingly most interviewees in this study taught this learner group. As such its findings may not apply to MO education in other groups such as pharmacists. We suggest that MO undertaken by physicians needs to focus on the roles and responsibilities of a generalist, a position supported by the views of physician workforce development planners in the UK [60]. The interventions and mechanisms required and involved in this education will likely be different to those of more protocol-based practice. Nonetheless, our PT does appear extensively aligned with similar work on medication review in pharmacists [61]. The interviewees in this study were all UK-based. Although the iPT underpinning this study’s PT was based on the international literature, and theorising was kept middle-ranged, our PT is liable to relate more to UK contexts (e.g. policies, regulators, healthcare and educational organisation) than to other countries.

The PT presented in this paper should not be considered a final product. Our PT requires refinement through further research to develop an increasingly granular understanding of this education. Discussion regarding the implementation of this work is also beyond the scope of this current study. Notwithstanding these limitations, RE such as this can be usefully used to clarify poorly defined phenomena and need not only be used to evaluate existing programmes [14].

### Future directions

Our theorising to date has been informed by published literature and interviews with experts. Next, we seek to incorporate the student voice. A series of MO educational interprofessional workshops, designed based on the PT of this study, followed by realist focus-groups, with their associated potential for sub-group analysis, is planned to test and refine theorising further. The refined PT resulting from this work will then be implemented within local medical programmes, evaluated and theorising further developed.

## Conclusions

Many patients, especially those living with MLTC, are not well served by the pervasive guideline-led approach to clinical decision-making; such a strategy frequently leads to PP with its associated risks and burdens [1]. Graduating physicians need the ability to practically integrate interpretive whole-person understandings of illness with the biomedical disease agenda to optimise their everyday clinical decision-making (1, 6). This ability can be exemplified in the practice of MO. The interpretive and integrative capabilities required during the clinical practice of MO will be developed throughout a professional lifetime, but an enabling culture and approach can be set in place during undergraduate training. This study builds on our prior realist review to refine an explanation of the workings of undergraduate MO education [8]. The explanation will be further developed via subsequent realist work. We highlight the practical priorities educators may wish to consider when designing or evaluating these much-needed educational programmes (Table 3). Understanding ‘what works, for whom, under what circumstances, how and why’ in MO education has been described as an ‘educational imperative’ because of its importance in tackling PP and, we suggest, its role as a practical gateway to developing learners’ ability in generalist, interpretive medicine [39]. Effective MO education therefore has clear potential to improve patient care, particularly in multimorbid patients.

## Additional Files

The additional files for this article can be found as follows:

10.5334/pme.2450.s1 Supplementary File 1. Transcript.

10.5334/pme.2450.s2 Supplementary File 2. Programme theory from the preceding review.

10.5334/pme.2450.s3 Supplementary File 3. Context-mechanism-outcome tables.

## Data Availability

The anonymised data of this study is available from the corresponding author upon request.
