# Supplementary material for: Understanding Undergraduate Medicines Optimisation Education and Its Importance; a Realist Evaluation
Source: Perspect Med Educ. 2026 Aug 25;15(1):647–62. doi: 10.5334/pme.2450 (PMC13523806; doi:10.5334/pme.2450)
Supplement: Supplementary file 1 [file pme-15-1-2450-s1.pdf]

Transcript

16 May 2024, 01:06pm

I started transcription

I 0:06
There we go. Should be recording now.
And so could I just start by asking, are you happy to have this interview recorded?

P 0:14
Yes.

I 0:16
Thank you. And so can I start with a really broad question and that's just about what do you see as the current needs in regards to medicines optimization, deprescribing education at the undergraduate level?

P 0:38
So that's a big question.
Do you mean by that what are the learning objectives at this stage? Or do you mean what is the ultimate outcome of why we need to be educating people?

I 1:00
And I mean, so a lot of people when they answer this question, do talk about the outcomes they'd like from the education.
But if you want to make it broader than that, you know if you had any other-

P 1:12
Well, OK, so I guess, you know, if we think about that and this is medical education specifically, we're not, we're not talking about other clinicians necessarily we're talking about Medical students.

I 1:24

Yeah, I'm focused on medical students, but I understand obviously that interprofessional learning is a key part. So if you want to talk about that.

P 1:30

Right. OK. OK.
Fine. Well, I mean, you know, if we if we just focus on, if we just think about the medical, you know, medical student education for now then.
I guess it's about for me there's the issue of how we integrate the use of medicines within clinical practise in its broader sense, so you it's quite difficult, I think, to disentangle medicines from everything else we do, which relates to diagnosis and, you know and shared decision making and subsequent care for patients. So medicines optimization I think it's important to understand that that's got to be integrated and at the moment I guess it is, but only because the use of medicines is touched on a relatively informal way by most clinicians. I think vast majority of teaching doesn't consider Medication review or, you know, as far as I'm aware and perhaps the geriatricians are perhaps the best, the best specialty who probably do consider Medication reviews and deprescribing. But I I'm expecting that vast majority of other ones don't you know because they're only interested to treat this condition, we have to add in drug X, drug Y, drug Z, you know? And the same goes for them. That's how organ based specialties work in the vast majority of cases.
General practise, you know similarly and obviously I'm a GP, so I'm coming from that perspective.
So for me, you need to teach students that medication reviews got to be an integral part of the process of practising medicine.
They also need to understand what how you should do, how medication reviews should or could be conducted.
That they should probably be patient centred, that they need to be addressing the patients concerns and priorities rather than necessarily just the condition ones, although there needs to be a prioritisation process there as well.
Obviously there's a whole host of sort of tick box things that you know that people like pharmacists, for example, are very good at doing, such as, you know, making sure that you're not having drug interactions and the doses are correct and there's nothing obvious missing. And there's nothing being left on the prescription that's no longer required.
Checking things like medication adherence. So those are the sort of fairly standard in

some ways relatively easy things to deal with, more challenging thing is to deal with that more holistic type big picture sort of stuff, particularly when you're coming to deal with patients with multiple medicines in the context of multi morbidity or in context of certain, you know health problems. I mean renal medicine being a prime example of one where there's so many other things going on and people end up on loads and loads of different drugs.

And then you're hoping that those students will be prepared when they actually come into foundation practise, for example to have an understanding of, you know, right, I need to review this person's medicines as part of the drug history. I need to review this person's medicines and decide, you know, are there opportunities for things to be tweaked, changed, improved and so on.

And understand what that process is and how it fits in with the broader clinical practise.

And then of course in the longer term, you hope that that leads to improvements in effectiveness of medicines in terms of clinical effectiveness, but also cost effectiveness in medicines, improved safety, and then ultimately you know that that filters down into to other clinical measurable clinical outcomes, whatever those might be.

I think at the moment one of the problems is that the medication use and clinical practise are, I mean certainly in primary care when I'm coming from, there's this idea that, well, this is something that should be done by pharmacists.

And it's sort of taken away from doctors as well. You know, this is something that pharmacists will do. That's fine. But I strongly believe that medics…

Yeah, they might not be quite so good at dealing with some of the technical aspects 'cause they don't know all the drug interactions and thing and the correct dose. It's not necessarily their fingertips, but because medicines are so inextricably linked to, you know, everything else that's going on with the patient. It's hard to separate it out, and it strikes me that, you know, doctors therefore need to be well equipped to be able to deal with medication reviews.

Particularly in the context of ageing populations those with multi morbidity and so on and then we can't rely on pharmacists to be doing all this. We need to have someone who has oversight and that that becomes Medics, ultimately.

So that’s a rather rambling response. I'm not sure if that answers your question or not, but anyway.

I 7:14

OK.

No, no, it does. So, so I mean, so you were saying there about you outlined quite a few of the outcomes that you were looking for. I mean, how might you design an educational intervention or programme that would allow the achievement of those outcomes. What would you include within that programme?

P 7:53

Well, I think the first thing to do of course you need to have an intervention that is going to work in the first place.

It's all very well designing some educational intervention. But actually, what are you educating people about? So you know, if you if we're gonna teach you how to do medication review. Then you need to actually have a medication review that works. So they need you need to have some understanding of the evidence base and the problem is that most of the sort of complex interventions that are out there at the moment in the context of polypharmacy, there's very little evidence that they work. They don't, you know.

There's Cochran review, obviously. It's been updated three times now on polypharmacy and older people and with very, very poor evidence. You know, there's a few trials that have showed stuff, but they're generally the certainty of that evidence is quite is very weak.

So the problem is you're trying to teach students about something where actually you're not entirely sure how to do it in the 1st place, because the evidence is missing. I mean we've done some research looking at you know medication reviews specifically in isolated medication review so thats outside the context of a complex intervention, and again, the evidence is just lacking. So despite the fact that usual practise is you should do a medication review, you know actually the evidence for what that medication review should look like, is missing.

And you know that that's problematic because you're wanting to teach students about medication optimization, but you're opening gambit ends up being 'and we don't really know how to do these or we don't know how to do ones that are effective' and that is very difficult I think to overcome. But ultimately you do need to have something. So you probably need to be pragmatic and say, OK, well, we don't really know. We don't have an ideal thing, but we think that reviews are probably a

sensible thing to do when you teach people how to do it, but you need to pick a model of medication review.

There are a number of models out there. I would of course push the Bristol medication review model because it's brilliant. But you know there's obviously other ones there.

Like the seven step model, which is obviously very established, you know WHO recognises it, it's used throughout Scotland, it is being updated.

So there will be changes to it in the latest iteration.

We heard that about three weeks ago.

I'll be tempted to avoid introducing models such as the no tiers model, which you know, although is used in the Welsh certainly advocate is, you know, underpinned by very, very little evidence whatsoever, in fact not really as far as I'm aware. You know, it was effectively a 2 pager in the BMJ many years ago. Someone came up with some nice acronym. I think that worked but there's very little. There's not even any behavioural, you know, theory underpinning it, other psychological theory underpinning it is just all this stuff, acronyms of things you might wanna think about asking.

So I think you need to focus on actually finding a review process, some sort of model that you might want to teach students to focus on. I think that's an important thing.

And then I think you probably you know this, similarly you could think about stuff for deprescribing models as well. Again, there's a few out there, but you might want to think about you know focusing on those sorts of things.

Something else I was gonna think of. Hang on.

It's called. It'll come back to me later, maybe.

So.

Yeah, I think is that is that answer your question maybe not completely. I think that's where you start with. You have to have some models and then in terms of how you teach them I think it's a mixture of, you know, theory and practise.

I think students need to have opportunities to apply that in practise they also need to have people who are able to actually observe them doing it and give them useful feedback, and that I think is really difficult because most doctors have no experience in how to do medication reviews.

Pharmacists do but pharmacists don’t have the same approach because they focus very much on technical aspects of medicines and stuff like that, and they therefore missed the bigger picture and we know that pharmacists are not good at doing

shared decision making when it comes to medication reviews. We'd hope that the medics would do that and also pharmacists don't deal with uncertainty very well, whereas medics are a lot more comfortable dealing with uncertainty. So, OK, so the students go off and they, you know, they're told to go off and do a medication review. And Mr Bloggs on the ward or, you know, in the GP surgery consultation or whatever it might be. And they do this review and then someone has to go over that with them.
And it's a GP or a nephrologist or whoever it might be, who doesn't actually have a clue how to do medication review themselves but probably capture some of the more holistic aspects of it. Or it's a pharmacist who you know could deal with the technical bits, but maybe doesn't do such a good job of assessing the more holistic care and patient centeredness side of things. So I think that's really difficult. And the same comes to teaching it, who teaches it, you know, because you can have a pharmacist teaching it, but they'll teach it very much from a pharmacist perspective or you can have a medic teaching it while you'll struggle to find them because there aren't that many medics you are familiar with how to use your medication reviews other than potentially geriatricians.
Or the occasional clinical pharmacologist like me.

I 14:39
So in terms of frameworks and schemas to undertake medicines optimization and structured medication reviews.
I mean, there's a theory that basically that there's quite a lot of cognitive load associated with doing medicines optimization and that maybe these schemas and frameworks give learners like a foothold, like an easy to understand foothold to, to then start practising and so in that way can reduce cognitive load, but the alternative theory is they could actually add to cognitive load because they're trying to talk to a patient and they're trying to think of these, you know, they're trying to keep these questions in their mind.

P 15:29
Yeah.
I think the answer to that is yes and yes.
So I think in my experience, most people, when they approach medication, you need some framework to work from 'cause otherwise they lose track of what it is they're

doing. Now people have different approaches. They may say you know, open up with a very open-ended approach where you say well, you know ‘what are your problems today, Mr Blogs?’ you know ‘what are the issues with your medicines you'd like to discuss with us?’. And you're very open-ended. And sometimes that works and sometimes it just runs into brick wall because you don't get useful response. Some people focus on it from a Medication perspective. So I'm gonna go through the ACE inhibitor and the beta blocker, then the calcium channel blocker, then the diuretic and then the whatever begins with E that I can't think of off the top of my head, but you know.
You know, and you just go through all the medicines in turn and tackle them one at a time.
Or you deal with it from a problems perspective. OK, let's talk about your angina. First of all, right, let's talk about your COPD. Let's talk about your epilepsy. Let's talk about your chronic pain or whatever, and deal with it that way.
So I think there's a whole host of different approaches and the models that you know models in some ways help because I think and if you look at the way things like 7 steps are structured and indeed our own review model and Bristol model and the idea was to focus on, ask the patient first of all and go from it that way, it's the only way. And the idea is to try and reduce the cognitive load in that sense because by asking the patient to prioritise what matters to them and focusing it that way, it becomes a lot easier.
You know.
If you got 20 different drugs, it's impossible to deal with that in 10 minutes or 20 minutes or even half an hour properly, so you can narrow it down by focusing on the priority, the patient priorities. So I think in that sense, the schema can help. And then certainly in the Bristol model, we've got a list of things that you have tries to jog the person's memory.
So things like adherence.
Over prescribing, under prescribing, you know?
Potentially unsafe prescribing things like that and things which you can sort of tick off.
Now the problem of course is occurs if you try to apply all of those to every single different medicine. Then I think you do run into problems with overburdening the condition with too many things to do and they oh, I was absolutely hopeless and that's very difficult. Whereas I think if you can, if the model emphasises focusing on a

particular patient priorities or indeed potentially important clinical priorities, you know the patient's coming in and complaining of pain in their arm, you know? And it feels as if pressing on my chest and yeah, I get a bit sweaty every time, it happens when I walk up a hill and you something. OK let's not worry about your sore toe. You know. Let's focus on the fact you seem to have angina. You know. So there will be clinical areas you know other persons on methotrexate and is you know developing you know some fibrotic lung or you know their marrow's packing in or whatever those are things, obviously with the clinical there is a need for clinical priorities to be at the forefront as well.
But actually my experience most of the time, the clinical priorities are pretty woolly, and often it's patient guided I think is helpful
So I don't have that answers your question about you know I think it is the case of there is extra cognitive load if you try and tick every single box on the pro forma, but certainly what we've said for the Bristol model, as we say, this is a, this is something you should use flexibly to suit your own needs, and it's there's a primer as a prompt for people, and I hope that then reduces cognitive load by, oh, here's something I can just flip back to every now and again. I don't have to do everything on it.
It's just a helpful reminder. I think the danger is if people do try to do everything on it, which is not the point of these things.

I 20:00
Yeah, and. And also how do you see the importance of the of the clinician understanding the patient as a whole person in context?
Because again, there's two arguments that it that could sort of add to self efficacy because the clinician would be able to understand the patient's goals and you know priorities in the in the medicines optimization, but it also could unearth a lot of other sort of difficult to deal with problems that could put the learner off that approach because you know because it just unearths so much that and they don't know how to deal with it, they find it difficult. So they just avoid that approach I mean.
How do you see understanding the whole person in context?

P 21:00
I think yes, you can unearth stuff, but it's important to emphasise well that we have, you know, a finite amount of time and stuff to deal with these issues. What do you think matters most at the moment with respect to your medicines? And there's a

variety of ways you might approach that in practise, but I think the first the first point you make is the is the is the pressing one.
Because you can deal with technical problems relatively easily, but it's a box ticking exercise and you probably don't even need a pharmacist to do it. You could probably do it with AI nowadays you know where you just tick off. You know, these drugs are the wrong dose and this drug might interfere with that and stuff like that, you know, but ultimately, you know you've got to understand what matters to the patient. And yes, the patient maybe is taking this drug at a dose which is not what the BNF says. If that's what helps manage their symptoms and that's why they feel well and they like to be on that, then fine. You know, you discuss the potential problems with that. But ultimately if it's what matters to the patient, it doesn't matter what the guidelines say in my view.
You know, it's about, it's about adapting to the needs of individual patients. I think more and more, you know, contemporary medical practise is moving down the line of you know where it's protocolized medicine.
And that doesn't work when you're dealing with complexity and uncertainty.
The point is, of course, we don't really know how to deal with complexity uncertainties. So the easiest thing to do is to ask the patient what matters most to them and use that to steer your way through, you know what's a very difficult maze of problems.

I 22:59

And what elements to this to the education might you add to maybe help students and deal with complexity and uncertainty? Is there any Element you would add?

P 23:14

I think you have to understand what complexity and uncertainty means in the first place because it's not just lots of stuff.
The complexity is different to something being complicated.
You know, complicated might be doing a difficult sum to work out the dose of drug that you've got to administer to someone, or involve a difficult process where you have to phone up lots of people to arrange for some imaging or whatever, you know, combinations of drugs what have you. Complexity is where there is this sort of non linear association between, you know, something going in, something coming out and it's unpredictable.

And it doesn't. It doesn't follow an obvious algorithm.
And I think it's important that students understand the, you know, challenges that they will face doing that in practise, and I suspect a lot of it's experiential learning. I'm not an expert on complexity and uncertainty by any means all though one of my PhD students is doing a PhD on the subject.
But, I'm sure there'll be probably some formal ways of teaching it, but I think a lot of it will probably be experiential learning. And you know on the wards or wherever it might be, where you actually encounter complex problems.
And a lot of it's you know case based learning and reflecting on the case with, you know, senior colleagues, experienced colleagues and so on.
So the exact content of that teaching, I'm not entirely sure about what is, I'm a bit more sure about is you probably need to do it with more experienced clinicians. I think deferring to younger people and less experience, I should say, physicians or medics, may not work as well because they just predominantly focusing on protocol driven, you know practise rather than you know knowing how to manage the case where you're not really sure. So I think there is something about experience amongst the educators.
And I have to say, I think the one specialty that does that well at the moment is geriatrics.
I guess general practise to a degree.
But I have to say that you know, as time goes on, I think I think General practise is becoming more and more 'we do this because that's what the that's what the guideline says', you know, rather than necessarily thinking out-of-the-box and stuff, which I think is I think, problematic when you think of the role GPs have as a sort of coordinator of care and things. So I think they defer too often to what specialists say. Which may not necessarily be the patient's best interest, because you know there's so many different specialties bombarding the stuff so that then contributes to people being on lots of medicines and so on. So I don't know. I mean, you know, again more experienced GPs, perhaps more comfortable about dealing with you know, uncertainty and doing things that aren't necessarily by the book. But I think we probably need to try and empower students to recognise that as a doctor you are in a different position and you need to be comfortable about, the Nice guideline says, on all of the guidelines it says 'this is a guideline you need to consider this in the context of the patient as a whole' or whatever the words are that it uses. You know, I think we need to encourage them to do that, not just teach them if X do Y which is

what currently I think often happens and certainly happens in some of the more certain medical specialties, I think where that, that can be more problematic than others.
They don't think about the big picture.

I 27:46
And in terms of you talking about having the experience for learning and then reflecting with colleagues that are ideally senior colleagues.
Do you think that that reflection and feedback needs to be multi professional. You know interprofessional or do you not think that's necessary?

P 28:10
I think that there are advantages to doing it that way. So you know I talked about the fact that you know you may not be able to find a GP, for example, who knows much about medication review, but they're good at, you know, patient communication and, you know, shared decision making, patient centred care and so on.
And you might be able to find a pharmacist, of course, familiar with the technical aspects of medicines, but can't do the other stuff. So there is something to be said about, you know, maybe doing the teaching in the context of having two people actually doing the teaching, you know, and actually they probably learn from each other in that sense that you know the medic then learns from the pharmacist and pharmacist learns from the medic. Now of course, you know that of course is difficult if you're going to do that in a one to one, to two basis. But you know that that's obviously difficult. But if you then know you can extend that of course to then multi professional learning where you have both teachers bringing their particular perspectives, but you also have pharmacists, pharmacist students and medical students learning together, for example, and the pharmacy students hopefully learn a bit about what the medics are saying and the medics hopefully learn a bit about what the pharmacist is saying. So you bring both sides, both bits of that along.
And we have used inter professional intervention as part of a polypharmacy trial, we we've had the pharmacist and the GP have dedicated time. They sit down together to do the medication review together and they've been different perspectives.
And the feedback from that has been overwhelmingly positive that the pharmacist benefit from the views of the GP and the GPs benefit from the views from the pharmacist. So both professions have been very, very positive about it. And I don't

see why you couldn't take that to an educational environment where both the teachers you have two teachers who have different perspectives and they can bring that, you know, to the table.
And perhaps, you know, perhaps if you're teaching medical students the medic is the one who leads the teaching and the pharmacist brings the pharmacy perspective as well 'cause they can add stuff in that the Medic doesn’t know about. If you're teaching the pharmacist, maybe you do it the other way round because ultimately pharmacists still need to be doing all the technical stuff. But you know, you make sure that the more holistic, patient, centred perspective comes into it as well.

I 30:41
Hmm.
And there's a theory that feedback in groups like that with continuity, so that the group and the faculty get to know each other can help with working out sort of safe boundaries for uncertain practise because the learners, because they get to know each other then they feel that they can sort of share their experiences in a more in depth way that they might not feel comfortable doing if they didn't know each other.

P 31:31
So I don't know about that from an educational perspective, but certainly that was one of the things that's come out of these this interprofessional reviewing process that we've been doing.
Where there is this degree of social support, where there's a bit of hand holding and you come across a difficult decision and it's, you know, maybe the GPs looking at this thing and thinking ‘well this has been prescribed by some clever hospital doctor, I feel uncomfortable about stopping it, but actually it really doesn't seem right to me’. The pharmacist is saying the same. They're viewing it saying, ‘well, you know, yeah, it's obviously it's an important doctor in the hospital has done this. But you know, this just seems wrong’. The dose is wrong or they could be causing this problem or that problem, whatever. Whatever. And actually speaking together the two together provide that reassurance to each other. You know you provide a crutch to each other, I guess to lean on that you say, ‘actually, yes, we can make a difficult decision here to to intervene’, you know?
And it's not just me on my own doing it so. So I think that social support thing is very useful. I don't see why that wouldn't work in an educational environment as well,

where you can, you know, if you have a safe space to share ideas and to recognise that there might not be a right and a wrong way to do this, and indeed that you might be potentially going against the norms. I think that that potentially does work and it doesn't necessarily have to be 2 professions. I think you could be just having, you know, the familiarity is probably important. So if you have two medics for example, you know working together to share ideas and both ideas back and forth and that's potentially useful. And I think the idea of familiarity, whether you know the person is useful as well. So although we've had a couple of different models for the intervention we've been testing, where we've had a pharmacist who's embedded in the practise already well known to the GP, they're a colleague, they'll meet them at coffee and so on. Because some practises didn't have a pharmacist, we've effectively dropped a pharmacist in. So they're sort of parachuted in and the relationship is different and obviously that working relationship, the strength of trust the ability to share difficult ideas and so on is not quite the same. So I think the former model where people know of a strong working relationship is potentially better, but I don't have any evidence for that.

I 34:08

And this is going off in a little bit of a tangent, but what do you think it is about geriatricians where they have a perception of greater self efficacy in doing this compared to GPs in general?

P 34:27

I think it's because they view themselves as experts in stopping medicines.
You know, and in dealing with uncertainty and in dealing with situations where there's perhaps more recognition that of the limits of medicine, what you can and can't achieve.
I think GPs sometimes struggle with that more. There's also an issue of hierarchy, almost certainly.
You know GPs viewing themselves as not at the same level as the as hospital colleagues.
So that's, that's certainly tricky. And I mean my own perspective, I trained as a clinical pharmacologist and worked as a consultant in hospital at the same time as working as a GP.
And I've sat on both sides of the fence, and it's very interesting to see it from those

two different perspectives.
Because you can kind of understand where the GPs are going from.
They don't feel empowered to make difficult decisions 'cause they don't wanna tread on anyone's toes.
And I think often the hospital physicians are often looking at it as well. We're expecting you to do this particular thing, why you're interfering and that often is, you know, I've certainly seen that from, you know, my hospital colleagues treating GPs in that way. Not all of them. And I think geriatricians are a lot better in that sense. They just don't seem to have that. You know, they accept uncertainty a bit more. Perhaps that's because there's no clinical trials in old people when it comes to drugs and stuff.
And obviously the fact people die when they're old and, you know, whereas the rest of us or, you know, the other specialties all trying to keep people going until the last minute. Geriatricians accept the fact you can't keep people going forever, you know. And they have a slightly more pragmatic approach. Maybe that's it.
And it's embedded in their psyche, of course as well, which is important. I think there is that sort of critical mass that you know, everyone in geriatrics thinks the same way. The problem is you can have this, you know, you can have some enlightened nephrologist like you thinking about or how you should be optimised, you know, medicines and stuff like that. But the problem is of all the other nephrologists are 'we need to add in this drug and that drug' and so on. You know? Yeah, I think actually nephrologists are possibly a bad example because I think you guys do deal with complexity a lot.
So, so cardiologists, let's go to cardiologists. They're awful, you know. You know, well, there's such and such a trial that's shown this. So we'll add in this one another trial we had in that one.
So it's this hierarchy and there's this culture of practise as well.
And then of course, when you try to…
You know, I think if you asked a cardiologist, for example, to review someone's medicines, it'll it'd be a completely different process. They don't think in a holistic or whole patient fashion. They're just thinking of this organ that's going hmpth, hmpth humpth in front of them.
You know, they have a very narrow, blinkered view, I think in many cases, I mean actually I'm tarring all cardiologists with the same brush. I know many who are very different to that. But I think the attitude and the way training is delivered is very

different. Cardiologists will not be, you know, will usually be just talking if they're teaching medical students, they'll be talking about ACE inhibitors and the trials that support their use and why they why they're useful and so on. And beta blockers. But they will probably think about them very much in isolation. They'll be very rare that they will be talking to the students in a more holistic way.

I 38:32

And do you think about the fact that if medicines optimization teaching is delivered at all, it's delivered right at the end of degrees, and that by that time quite a lot of students might have quite a biomedical perspective on illness and do you think that sort of factors in?

P 38:59

I don't know. I don't know the answer to that, I think probably, you know, there'll be a lot of variation. I think it's forgotten about. So I mean we used to teach this in <<name removed>>.
We would teach medication reviews in year two, I think.
Possibly right at the start of year three, but it was so early, they didn't really understand how it worked and it probably had all forgotten about it, you know, by the time they've done all the other stuff. So then the alternative is then you chuck it at the end, but then at that point, as you say there is, you know, they've got this ingrained approach as to how they're going to do it, and 'oh God, we've been taught something else'. Now it's too late to change that practise. So I think you have to do it throughout from the very beginning to the very end.
You don't make it tokenistic. You make it an integral part of practise and this comes back to this whole idea of making holistic, making it patient centred, it is not just about dealing with a list of medicines and it's not an extension to doing a drug history. It's about understanding how those drugs affect the person in the wider sense.
From a social perspective, from a medical perspective.
But it has to be embedded throughout the whole course. I think that that's the critical bit. I don't think you can stick it at the end or at the beginning because it'll either get forgotten or it'll be too late.

I 40:33

Hmm. So how do you see this teaching sitting in relation to the rest of the curriculum? I mean, you're touching on that already, but are you able to talk a bit more detail on that?

P 40:48

I think you have to do it.

From so I think you should do it from the very beginning. You need to explain the purpose of it at the start, and obviously they won't understand all the technicalities of it and things like that that start. But you need to say that this is something you should be considering for all patients every time you see them. Really, you know in most circumstances, or at least at least once during their during their hospital stay, you know at least once if they haven't been seen for a while, they present with a new problem. And in general practise for example you know you need to do this in a regular basis. You drill that into them for the beginning.

You deliver it on a regular basis throughout the curriculum, so you know, year one, year two, year three-year four, Year 5.

You can add one or two bits in as you go, I guess, and the and the sophistication of the way you're doing the teaching will increase as you move on.

But they have to. It has to cover most of the basis from the very beginning. You have to make it evident this is normal practise.

And not a little add on tokenistic gesture, which I think it gets treated as at the moment.

I 42:02

And do you see this as obviously a core part of prescribing teaching or do you see this as weaving into, you know, communication skill teaching and then you know, so it would weave between different themes and you'd pick it up?

P 42:17

I think I think it's both. I think it's going to be both. So I think you know call it a spiral theme or you know it's both horizontal and vertical in some ways.

So you know, they need to understand that if you're starting a new drug, then it will be important to review that drug and that review should be done relatively early on in the process to make sure the person's not getting an immediate side effects they need to, you know, address issues around monitoring and stuff, they need to make

sure, of course you're starting that medicine in an understanding of all the other drugs that the person's on and their clinical context, you know they got renal impairment or if they got, you know liver failure or whatever, is it going to cause problems with falls because there's an elderly person, blah blah blah. So you know, that applies to new drugs, but it a drug, it applies to assessing someone who's feeling unwell. You know, who's maybe had a fall, for example. That's a cue to do it. And remind me of the question.

I 43:21

And it was about whether it would run all the way sort of vertically through a prescribing-

P 43:30

Yeah. So I think so. I think I've answered that in some ways. I think you know if you've got, you can think about it from if someone's got a new medicine or a change in medicine, there's a specific issue in relation to medicine, then you need to be thinking about doing your reviews. So every time you're introducing new drugs, you should be talking about, well, remember to start this new drug and remember about your medication reviews when you're going to do that, you know, and you can send people away to practise that. And similarly, if they're coming in with a clinical problem, falls or a new diagnosis of angina, or, you know, dementia or whatever it might be. You need to be thinking about medication review. You know, it might be a social problem that the person is presenting with. How are you going to deal with that from the medicines point of view, what happens if the person can't get to the chemist to pick up their pills or they, you know, can't read the pills because they don't speak English or they're illiterate or things like that. So there are all those sort of issues to consider as well.

Now who has ownership of that is a difficult one. So it might be that the ownership is with- You know what? I'm clinical pharmacologist, you know, do medical education around, you know, medicine. So I'm one of them, but there's hardly any of us in the country. So we can't exactly do this. It's very difficult.

You know, geriatricians potentially, but it's important to emphasise that medication reviews are not just in old people. They're also in young people as well. So you know, those are two specialties that could do it.

You could get the organ based specialties to recognise it more, but that's a real

challenge. So I don't think you know I'm not sure one person necessarily has overall ownership of it. I think everyone needs to have ownership of it. I guess it will depend on individual curriculums in terms of how they're based. So if there's a particular theme. So for example, I was the lead for pharmacology and therapeutics teaching in <<name removed>>. I have the same role in <<name removed>>, you know, so it's either responsibility for that, as it were.
But then it often gets divested to some other particular person who does that teaching, but someone probably needs oversight of it to make sure it fits within the curriculum.

I 45:56

We haven't talked about it yet, and that's assessment.
In the literature, a lot of these educational interventions don't have any summative assessment and sometimes not any assessment at all when you know, and it seems reasonable to think that that would affect how students, you know, value the education and engage with it, I mean.
Do you have any thoughts on how this should be assessed?

P 46:32

Well, obviously in some ways that the prescribing safety assessment already addresses some rather narrow aspect of it. So it picks up on issues around dosing and safety issues and monitoring and things like that. Of course, what it doesn't do is address holistic aspects of it. I think those can be done, you know assessed.
It's quite difficult to assess them in things, you know, things like multiple choice and stuff like that. I guess. I think OSCEs are an obvious way of assessing it.
And practise based, you know, assessments.
And, you know, bedside assessments or whatever are the best ways of probably doing it. Now whether that's summative, whether that's formative. I'm not sure. I think it probably should be some sort of summative aspects to it.

I 47:34

Yeah. And do you think that the trend in medical education towards increasingly reliable assessment tools like multiple choice questions and single best answers and whatever is moving medical learning away from nuance and therefore-

P 47:56

Yes, yes, yes, very much. So. I've been asked to write questions on polypharmacy in the past for mcqs and it's a bloody nightmare because the questions you ask are pointless and you know, we all know there's all these rules and how you have to set up the alternative answers, you know, and they have to pass the cover test.
You can't do that with polypharmacy and with uncertainty you just can’t. It just does not fit the bill. So the questions you end up asking are just not actually addressing, you know, anything particularly useful.
You end up seeing stuff like you know, what proportion of people are on, you know, seven or more medicines aged in their 70s, useless facts which you know, actually don't really make you a better doctor necessarily. I don't think Mcqs can ask the questions you need to when it's coming to dealing with uncertainty, you just end up with basic fact checking for specific things like, you know, which of the following is the best test for monitoring someone's methotrexate long term or you know.
You should check the digoxin level at 8:00 AM. You know, four pm, 8:00 PM, midnight. Whatever. You know those sorts of things which are I don't think capturing what you need to here.

I 49:15

Hmm.
Yeah. OK. Great. Yeah. Thanks so much. That's really useful. I think I'm going to enjoy looking through all that and trying to analyse it. I'm just thinking if there's anything more.
Just as a bit more of a general question.
What would you want as an educator to see as the outcome from a study like mine?

P 49:48

I would like to know how we embed or encourage people to embed this subject matter within the curriculum.
At the moment I just I think it just doesn't happen. People forget about it and we need to understand where it should fit and how it should fit and how you actually.
That that's for me what would be most useful I think. I mean, people always have different perspectives in terms of what actual material you're going to teach at the end of the day.

But I think it's about, I mean we've got incredibly busy curricula which have so many different people, you know, saying I wanna teach this. I wanna do that and so on. Well, I'm exactly the same. I think that we should be teaching medicines optimization much better. But how do I make sure that that you know that my voice is heard amongst all the other specialties who are clamouring for their particular area to be integrated.
So I think that that for me that would be really useful to understand how that, how that be achieved, how people think it should be achieved.
Not sure if that's what you were expecting to hear.

I 51:14
No, no. Yeah, no, it is that that makes sense.
Great. Is there anything more you'd like to add, that you feel you haven't said? Before we wrap up.

P 51:29
I don't think so at the moment. I think you know it's clearly a challenging area and I could probably talk for hours about this but your transcription costs won't cover it. I suspect Richard.
Yeah, I think we've you've covered some of the key points you know you've obviously recognised issues around the issues around interprofessionalism and you know issues around you know we've talked about issues around things like hierarchy and the differences between very focused reviews compared with more holistic ones. So I don't think I think there’s nothing immediately springs to my mind. I think kind of most of the broad things but I'm happy for you to come back to me if you want to. To go over anything again.

I 52:25
Oh, thank you that that would be really good. Well, thanks very much. I'll stop the recording.
