# Supplementary material for: Understanding Undergraduate Medicines Optimisation Education and Its Importance; a Realist Evaluation
Source: Perspect Med Educ. 2026 Aug 25;15(1):647–62. doi: 10.5334/pme.2450 (PMC13523806; doi:10.5334/pme.2450)
Supplement: Supplementary file 2 [file pme-15-1-2450-s2.pdf]

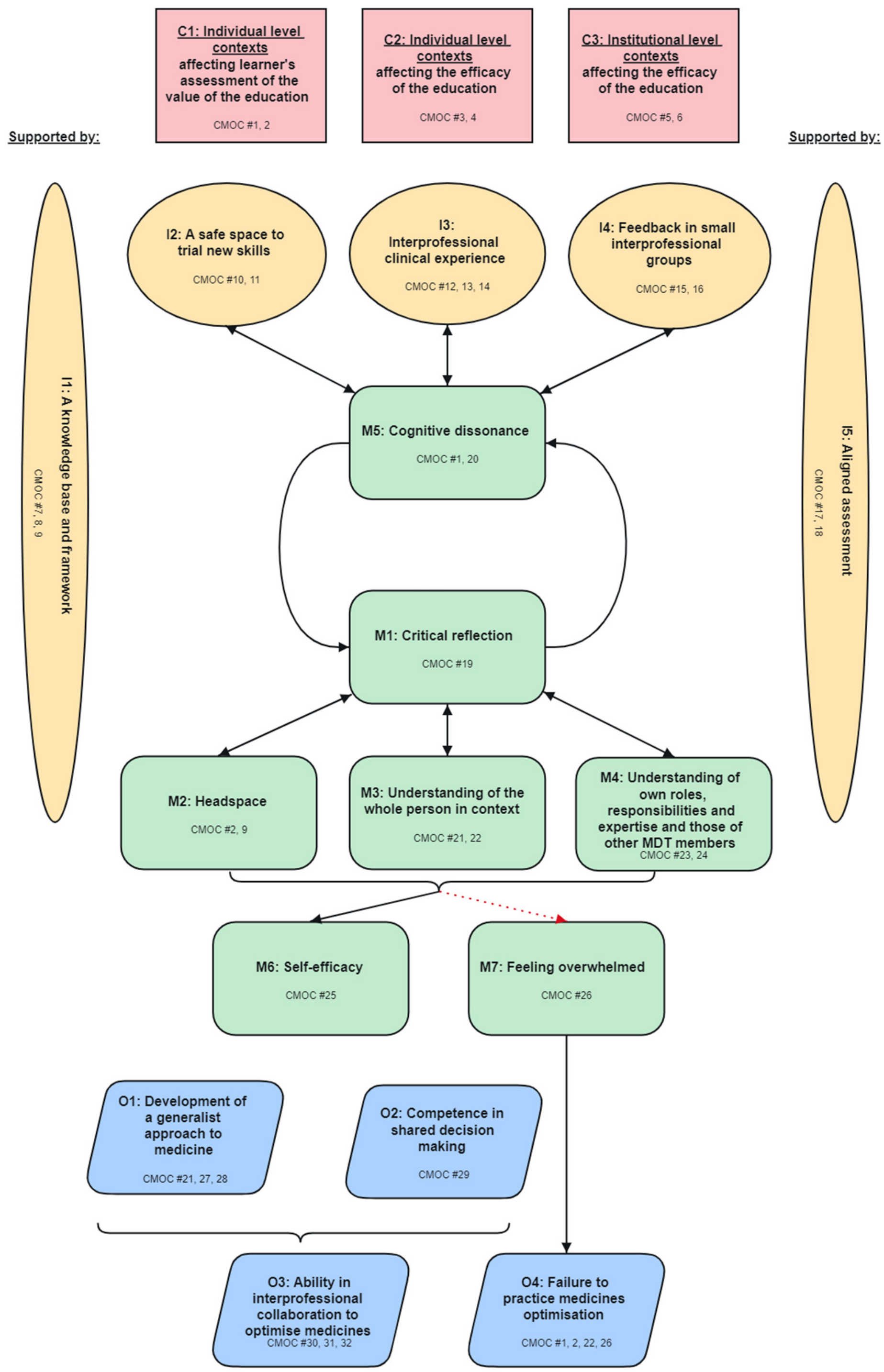

Figure 1: The programme theory (PT) resulting from our realist review, which preceded this study. This PT acted as the starting point (i.e. initial PT) for the interview series. MDT: Multidisciplinary team, CMOC: context, mechanism, outcome configuration.
