# Supplementary material for: Understanding Undergraduate Medicines Optimisation Education and Its Importance; a Realist Evaluation
Source: Perspect Med Educ. 2026 Aug 25;15(1):647–62. doi: 10.5334/pme.2450 (PMC13523806; doi:10.5334/pme.2450)
Supplement: Supplementary file 3 [file pme-15-1-2450-s3.pdf]

Table 1: CMO configurations relating to prioritisation, perceptions of value and engagement. MO: Medicines optimisation, UG: Undergraduates, MCQ: Multiple choice questions, MBBS: Batchelor or Medicine, Batchelor of surgery (primary medical degree), MLA: Medical licensing assessment.

CMOC #	CMOC	ILLUSTRATIVE QUOTES
**3**	Medical schools operate in highly resource constrained environments (C). If MO competencies are mandated in regulatory body graduate competencies (resource M) medical schools are likely to perceive increased value in MO (response M) and direct activity and resources towards MO education programmes (O).	-4-11: *You only have to look at the activity that goes on when a new assessment is created. It certainly was case of the prescribing safety assessment, where new posts were created in medical schools to help people sort of develop a little bit more in that direction. Student groups were set up in various universities that that you could see. And it's happening now with the MLA.*<br><br>-4-11: *It's additional resource to actually set all of this up and in a really challenging resource challenged environment, which almost everybody running a medical school tells me they're in.*
**4**	Assessment in medical schools in the UK is becoming increasingly standardised and dominated by the use of highly reliable assessments, predominantly MCQs (C). A culture of assessment that suggests scenarios have only one correct answer (resource M) may drive learning in medical students away from an acceptance of nuance in clinical practice (response M) which may inhibit ability in complex, uncertain tasks such as MO (O).	-4-12: *The way that undergraduate training is at the moment and with MLA on the way, it's just a really difficult space in which to be doing this.*
**5**	MO is likely to be best taught in small groups with high student to staff ratios (C). MO programme faculty that insist upon highly resource intensive methods of delivery (resource M) may be perceived as impractically idealistic by programme directors (response M) and the MO theme may not be engaged with meaningfully as a result (O).	-4-12: *We get criticised because we have very high, for most learning experiences, tutor to student ratios and also the tutors are all clinicians. So, they're expensive. I realise this is not going to be something that people are going to like as a model of educational learning.*<br><br>-4-11: *I just don't think there's ever going to be enough resource, and particularly clinical resource, and staff time to do this all based on small group teaching and discussion of cases.*
**6**	MO needs to be visible to gain institutional traction and resources (C). MO programmes that explicitly label activities and learning as pertaining to MO	-4-25: *Well, we don't have a specific module that mentions that so it must not be that important.*

	throughout the MBBS (resource M), may clarify the central nature of MO in medical programmes (response M) and boost learners and faculty engagement with the learning (O).	-5-16: *You have to make it evident this is normal practice and not a little add on tokenistic gesture, which I think it gets treated as at the moment.*
**7**	If MO were meaningfully incorporated into the MLA (C), this increased priority and visibility of MO (resource M) may trigger a concern in programme organisers for learners to meet expectations (response M) which may be leveraged to boost resource allocation to MO programmes (O).	-4-252: *For example in the MLA and in the prescribing safety assessment and such assessments like that where we've got a national sort of collaboration in terms of what we're going to assess and what's going to be in there, if we have that kind of leverage to get medicines optimization within there in one way or another, that would push all medical schools to make sure they addressed it in a better way.*
**8**	MO education in the UG setting has no agreed learning outcomes or methods for delivering this education (C). Efforts to produce a standardised set of outcomes in MO for UK UGs (resource M) may trigger a perception of the importance of MO within medical educators (response M) which could lead to the meaningful incorporation of MO into the MLA (O).	-4-11: *I think learning objectives need to be agreed and clarified. We can't learn everything about these kind of topics, but it's kind of really helpful if people have got together and actually worked out what it is that is required. I think in terms of knowledge, skills and other attributes, if you are going to be someone at foundation level who can start to contribute to the medicines optimization process, then there must be some basic skills there.*
**9**	In a healthcare system focussed on standardisation and speed in outcomes (C), MO programmes that focus on nuance and outside of guidance individualisation of care (resource M) may be perceived as idealistic (response M) and may not be valued by educational decision makers (O).	-4-12: *We've designed a system that's so obsessed with standardising care, with reducing everything down into little units that we can standardise and push through, ideally as quickly as possible, that actually we prevent the rats on the wheel from delivering anything close to those people standing there, saying 'you need a personalised optimization of medicines'. Great, those two are incompatible.*
**10**	When MO in primary care settings is increasingly delegated to pharmacists (C), MO education for UG medical students (resource M) may be considered irrelevant by educational decision makers (response M) and result in them not prioritising the education (O).	-5-16: *I think at the moment one of the problems is that…I mean certainly in primary care when I'm coming from, there's this idea that, well, this is something that should be done by pharmacists.*
**11**	A desire for maximally reliable methodology drives much of assessment design in medical schools (C). MO outcomes that prioritise assessment reliability above validity (resource M) may result in an inappropriate lack of nuance in MO learning (O) via	-5-16: *I've been asked to write questions on polypharmacy in the past for MCQs and it's a bloody nightmare because the questions you ask are pointless. You can't do that with polypharmacy and with uncertainty, you just can't. It just does not fit the bill. So, the questions you end up asking are just not actually addressing, you know, anything particularly useful.*

	the tendency in students to learn in keeping with the requirements of assessment (response M).	
**12**	Medical schools must have reliable, defendable and feasible assessments (C). MO programmes that fail to engage with these requirements in assessment (resource M) may be perceived as 'out-of-touch' by programme directors (response M) and result MO programmes being assessed formatively only (O).	-4-22: *You have to have a reliable exam with a decent alpha coefficient. You just do, so you don't get sued.*

Table 2: CMO configurations relating to intervention elements; prerequisites, a place to trial and reflect and a community of practice. MO: Medicines optimization, UG: Undergraduate, MDT: Multidisciplinary team.

CMOC #	CMOC	ILLUSTRATIVE QUOTES
**1**	Universities make active decisions in their student recruitment priorities and widening participation activities (C). A range of lived experiences in a group of discussants (resource M) may allow a fuller appreciation of the whole	4-23: *If you have certain goals for medicine optimization really you should be choosing the candidates who will be responsive to those goals. So, there's a lot to be said about that and I would couple that with widening participation. That with a with a wider pool of*

	person in context (O) via the challenging of assumptions and triggering of critical reflection in learners (response M).	*students from different backgrounds, you're more likely to get medical students and doctors who appreciate people's backgrounds in an informed way.*
**2**	Learners are more likely to value and engage with learning if they feel it will be key to their future roles (C). MO education that sets out the roles and responsibilities of learners, in regards to MO, from early in the course (resource M) may result in learners integrating MO into their routine practice (O) via a perception of preceptors expectation's in MO ability (response M).	4-25: *I think if students saw that medicine's optimization carried similar weight to other things, then I think again they would be more likely to go away and do it because they recognise it's an important part of their roles.*
**13**	Leaners are likely to prioritise the acquisition of clinical practice patterns they observe regularly (C). Programmes that undertake clinical staff development in making MO practice explicit and central to consultations (resource M) may reinforce the relevance and importance of MO to learners (response M) and promote engagement with the learning (O).	9-20: *If you did the tranche of staff development so that the tutors knew the importance of medicines optimization being an absolutely core part of the discussion around a consultation. Because that's embedding it in context situality theory with an experienced other.*
**14**	Within the clinical placement component of MO programmes (C) experience where learners see members of their own profession routinely optimising medicines (resource M) may trigger an appreciation of the expectation and responsibility in the task (response M) and lead them to prioritise MO learning going forward (O).	4-25: *I think you'd want to almost cherry pick a little bit so they can see them doing this as part of their day job, so you've got that kind of role recognition again that would reinforce that as well, right. You know 'these people are really senior in my profession, they're doing it, right I should definitely be doing it'. 'And now linking what I'm doing at university, I can see now how that's working out in practice'.*<br><br>4-25: *I often think one of the deficits we have is people think 'I'm not going to have to do that later' or 'I'm not going to take the rap for that later'. So, you prioritise in your head, don't you? What you're going to learn about, what you're not going to learn about, based on what's useful for you later or what might save your bacon later.*
**15**	The practice of MO is more nuanced and 'greyer' than it appears in theory (C). MO programmes that provide opportunities for extended meetings with real patients with polypharmacy (resource M) may trigger an appreciation for	5-16: *It doesn't follow an obvious algorithm. And I think it's important that students understand the challenges that they will face doing that in practice, and I suspect a lot of it is experiential learning.*

	the non-linear relationship between inputs and outputs in MO practice (response M) and prepare learners for complex real-world practice (O).	
**17**	Students prioritise learning based its perceived requirement in practice (C). MO programmes that proffer a level of responsibility over patient care (resource M) may trigger a perception of risk and importance in MO (response M) and lead to greater engagement with the learning (O).	4-22: *And then when you first get into the car and drive around the car park, yeah, there's an element of concern. But if the carpark's empty, the fear level is quite low. But very, very much when you take the car out of the carpark onto the main road and other road users are suddenly around, I think everybody goes 'oh' and they get their game face on and start to make sure they do it right. And I think pre-prescribing does that.*
**18**	The clinical practice of MO infrequently provides positive feedback to practitioners (C). MO programmes that provide contemporaneous feedback on all MO practice (positive and negative) (resource M) may trigger a behavioural change response in learners (response M) and lead to effective learning on the subject (O).	4-23: *If you give it to them three seconds later then that actually has a positive effect, and sometimes it seems as if being wrong and being corrected immediately is better for long term recall than being right first time. So, there's something about that experience of being immediately guided. That they were incorrect and what the correct answer would have been actually seems to reinforce the neural pathways more than just getting it right first-time round, which is very interesting.*<br><br>4-25: *So people kind of want some feedback, you know, and from a behavioural perspective, this is about reinforcing good behaviour because at the moment you spend hours doing a medication review and you don't ever really find out what happened or whether it was any good.*
**20**	To be effective, MO decisions must be made with a good understanding of the patient in context (C). MO programmes that provide opportunities for interprofessional discussion around a patient (resource M) may trigger an appreciation of the different perspectives on the patient (response M) and lead to a more realistic view of the whole patient in context (O).	4-23: *That with a wider pool of students from different backgrounds, you're more likely to get medical students and doctors who appreciate people's backgrounds in an informed way.*
**21**	Due to curriculum time restrictions learners may have very few opportunities to practice MO (O). MO programmes that give feedback on performance but not opportunities for further practice (resource M) may fail to consolidate that	4-30: *And this is the nugget through all education; you can give the most wonderful feedback or feed-forward or whatever, but if students don't apply it, reflect on it, then it is pointless.*

	learning (O) due to learners not being able to repeatedly reflect on concrete experience (response M).	
**22**	MO practice is not heavily underpinned by evidence and many learners feel vulnerable in uncertainty (C). MO programmes that teach learners with widely used implicit frameworks to approach MO (resource M) may promote a feeling of confidence in learners (response M) and allow them to more readily practice MO (O).	5-2: *I think that's kind of building in your quality assurance, and I suppose the tools are a way of integrating evidence-based practice without needing to carry all the evidence in there around in your head.*
**23**	MO requires knowledge of the patient and of their medications (C). MO programmes that attempt to teach MO prior to teaching on clinical pharmacology and therapeutics (resource M) may fail to engage learners (O) because of learners' inability to visualise its practical application (response M).	4-22: *It's a more complex skill, requires a lot more general pharmacological and therapeutic knowledge of the students, so it has to come later on the course.*
**24**	Much of UG medical training is taught and assessed without explicit appreciation of nuance (C). MO programmes that provide HCP discussion of different approaches to tackle complex MO cases (resource M) may trigger confidence in learners (response M) to engage with nuanced MO decisions in complex patients (O).	5-16: *If you have a safe space to share ideas and to recognise that there might not be a right and a wrong way to do this, and indeed that you might be potentially going against the norms. I think that that potentially does work, and it doesn't necessarily have to be two professions… just having the familiarity is probably important. So, if you have two medics for example working together to share ideas and bounce ideas back and forth that's potentially useful.*
**25**	MO practice varies significantly between individuals (C). MO programmes that provide a space to trial new tools and techniques (resource M) may trigger a perception of self-efficacy in learners (response M) and improve chances of MO practice on clinical placements (O).	5-2: *It's about kind of trying those for size and deciding what's going to work for their own particular style of consultation and the environment that they're in. And I think part of that as an undergraduate is the opportunity for those experiences so that you can go through that process of what's going to work best for you as a practitioner because not all doctors are the same, are they?*
**26**	MO practice is a complex, multi-step skill developed over time (C). MO programmes that provide MO activities within novice learner's zone of proximal development (resource M) may trigger a sense of achievement in learners (response M) and result in motivation to further practice MO (O).	4-23: *So your zone of proximal development is the amount that you can reasonably learn. And as a novice, that's quite small. And then you gradually expand that bit by bit. Trying to make it too complicated too soon means that students may well learn nothing at all.*
**27**	UG students have large quantities to learn and may forget skills not used routinely (C). MO educational interventions	4-23: *You're raising the arousal level a little bit and therefore it helps encode the information, it helps it pass from short term to*

	that use concrete experience to heighten emotion in learners through discussion (resource M) may more readily trigger reflection on the learning (response M) and lead to lasting, integrated learning on the subject (O).	*long term memory.*<br><br>5-2: *There's no way that you can connect with somebody on an emotional and an intellectual level in the way that actually asking people who have used multiple drugs for multiple conditions can.*
**29**	IPL allows the integration of learners from different professional groups at different stages in their training (C). MO educational interventions that facilitate the demonstration of the visible expertise of other members of the MDT (resource M) may trigger respect in learners (response M) and lead to them valuing the skills of the MDT ongoing (O).	4-23: *When our former nurses took their first progress tests, they massively outperformed these kids from school with 3A's at A level. And of course, the nurses were also outperforming these students massively in clinical skills. So instead of the nurses being looked down upon, they were by definition looked up to. The assessment methodology had made a huge difference by valuing their previous experience.*
**30**	In medical programmes delivered on a foundation of the pre-eminence of person-centred practice (C), MO education that highlights scenarios where medical students are unable to deliver a tailored, person-centred solution to a problem alone and require multi-professional input (resource M) may trigger appreciation of multiprofessional expertise (response M) and foster more positive attitudes towards interprofessional collaboration in MO (O).	4-12: *That in itself is a really important learning outcome, because then the facilitator is 'OK, well, who else do you need in the room to be making that decision?' because the patient's flagged up an issue for you, you have realised that you don't have the skills, the knowledge, the data, whatever, to make that tailored optimised decision with the patient. So what do you need to make that happen, rather than either shutting the conversation down and just sticking with the guideline or forcing the patient into something they don't want?*
**31**	Medication review proformas can be used as the basis for MO (C). MO education that focusses on explicit and lengthy medication review checklists as the basis for all MO (resource M) may lead to learners perceiving MO as a protocolized activity (response M) and disengaging from the learning (O).	5-16: *Now the problem, of course, occurs if you try to apply all of those (criteria) to every single different medicine. Then I think you do run into problems with overburdening the student with too many things to do.*
**33**	MO education delivered on the background of generalist, person-centred practice (C) that encourages the use of implicit MO frameworks focussing on patient priorities (resource M) may free up headspace in learners (response M) and provide clarity of purpose (O).	5-16: *As we say, this is something you should use flexibly to suit your own needs, and it's there as a primer, as a prompt for people, and I hope that then reduces cognitive load by (allowing the learner to think) 'oh, here's something I can just flip back to every now and again. I don't have to do everything on it'. It's just a helpful reminder. I think the danger is if people do try to do everything on it, which is not the point of these things.*

		5-16: *The point is, of course, we don't really know how to deal with complexity, uncertainties. So the easiest thing to do is to ask the patient what matters most to them and use that to steer your way through what is a very difficult maze of problems.*
**36**	MO education is only one component in a medical curriculum (C). If learners find the culture and practice regarding MO on their placements to be out of keeping with their teaching (resource M) they are likely to reject that learning (response M) and fail to practice MO (O).	4-25: *I guess there is a risk that if we send them out with some placement objectives about doing some of this stuff and then they then don't see it happening in practice, or people in practice saying 'Oh no, that's far too risky. Leave it alone' or 'we don't have time to do that' or 'that's not a priority'…They might find that, obviously counterproductive.*
**38**	MO practice cannot be protocolised and evidence to support decisions is generally absent (C). MO programmes that prioritise the grey-scale, in-context nature of clinical decision making (resource M) may trigger a perception of permission to step outside of guidelines as necessary (response M) and facilitate a generalist approach to medical practice (O).	4-12: *Sticking your neck out is actually part of what being a professional is about. You're not just a technician following the rules.*<br><br>6-24: *So I think we often get to the point that there's not one right way, but it allows us then to talk about our thought processes, the steps that we've taken to get to that decision, and they can then have some extra things to think about if they're approaching that decision…and a broader view that can help them then to make those sorts of decisions.*
**40**	Work-place culture and fear of criticism comprise greater barriers to MO practice than lack of knowledge (C). MO programmes that promote interprofessional collaboration in MO (resource M) may promote a sense of a community of practice in learners (response M) to overcome fear of acting outside of guidelines (O).	5-2: *But I think also in terms of confidence to deprescribe, I think if you know that your line of thinking is aligned with what the person wants, and what a number of your colleagues is thinking, you're more likely to be taking the step to discontinue a preventive treatment than if you feel like you've got to make a decision alone.*

Table 3: CMO configurations relating to primarily to outcomes; an interprofessional generalist community of practice prioritising routine medicines optimization. MO: Medicines optimization.

CMOC #	CMOC	ILLUSTRATIVE QUOTES
**22**	MO practice is not heavily underpinned by evidence and many learners feel vulnerable in uncertainty (C). MO programmes that teach learners with widely used implicit frameworks to approach MO (resource M) may promote a feeling of confidence in learners (response M) and allow them to more readily practice MO (O).	5-2: *I think that's kind of building in your quality assurance, and I suppose the tools are a way of integrating evidence based practice without needing to carry all the evidence in there around in your head.*
**25**	MO practice varies significantly between individuals (C). MO programmes that provide a space to trial new tools and techniques (resource M) may trigger a perception of self-efficacy in learners (response M) and improve chances of MO practice on clinical placements (O).	5-2: *It's about kind of trying those for size and deciding what's going to work for their own particular style of consultation and the environment that they're in. And I think part of that as an undergraduate is the opportunity for those experiences so that you can go through that process of what's going to work best for you as a practitioner because*

		not all doctors are the same, are they?
**34**	Learners and their supervisors are frequently fearful to make outside of guideline decisions required in MO practice (C). MO education that promotes the perception that optimisation of medicines using generalist principles is core to the role of all doctors (resource M) may promote a perception of confidence and security in MO decisions (response M) and increase the likelihood of routine MO practice (O).	5-16: *Geriatricians accept the fact you can't keep people going forever, you know. And they have a slightly more pragmatic approach. Maybe that's it. And it's embedded in their psyche, of course as well, which is important. I think there is that sort of critical mass that you know, everyone in geriatrics thinks the same way.*<br><br>4-12: *But I just think we need to get back to the idea that it's a professional responsibility if you like... Sticking your neck out is actually part of what being a professional is about. You're not just a technician following the rules.*
**35**	The meaningful practice of MO cannot be protocolised, there is limited scope for practitioners to draw on evidence to support their decision making (C). MO education that promotes, as a key task, gaining an understanding of the whole person and their priorities in context (resource M), may provide clarity of purpose to learners (response M) to act as a guide to undertaking MO routinely (O).	5-16: *The point is, of course, we don't really know how to deal with complexity, uncertainties. So, the easiest thing to do is to ask the patient what matters most to them and use that to steer your way through what is a very difficult maze of problems.*
**39**	Solutions to complex MO problems cannot be amalgamated from existing protocols (C). MO programmes that place advanced generalist clinical reasoning as the central principle of decision making (resource M) may trigger creativity in learners (response M) and enable tailored context-dependant solutions to MO problems (O)	4-12: *You cannot optimise medicines, with your definition of person centred, safe and effective use of medicines around the person- what Keith Ridge said, you cannot do that unless you have the skills to create knowledge in context. Critically create knowledge in context, use it and critique it and change it.*<br><br>4-12: *Medicines optimization is about taking it even further back than thinking about medicines and it's into advanced generalist clinical reasoning. Clinical decision making. Interpretation of illness and so on and so forth.*
**40**	Work-place culture and fear of criticism comprise greater barriers to MO practice than lack of knowledge (C). MO programmes that promote interprofessional collaboration in MO (resource M) may promote a sense of a community of practice in learners (response M) to overcome fear of acting outside of guidelines (O).	5-2: *But I think also in terms of confidence to deprescribe, I think if you know that your line of thinking is aligned with what the person wants, and what a number of your colleagues is thinking, you're more likely to be taking the step to discontinue a preventive treatment than if you feel like you've got to make a decision alone.*

**42**	Fear of litigation in making out of guideline decisions may inhibit medical graduates from practicing MO, resulting in deskilling (C). MO programmes that provide opportunities to develop shared decision making skills (resource M) may provide a perception of security in decision making (response M) and increase the likelihood of MO practice after graduation (O).	8-16: *And the other thing is that shared decision making can reduce the fear of litigation and it's also really important to have pharmacists and doctors doing it together.*
